# Expert Panel Consensus Guidelines of the German Society of Neuroradiology on the Use of Magnetic Resonance Imaging in the Diagnosis and Monitoring of Multiple Sclerosis

**DOI:** 10.1007/s00062-026-01670-4

**Published:** 2026-05-26

**Authors:** Mike P. Wattjes, Carsten Lukas, Sönke Langner, Hagen H. Kitzler, Michael Scheel, Andreas J. Bartsch, Benedikt Wiestler

**Affiliations:** 1 Dept. of Neuroradiology, Charité - University Medicine Berlin, Berlin, Germany; 2 Institute of Neuroradiology, Ruhr University Bochum, Bochum, Germany; 3 Enradia, Greifswald, Germany; 4 Department of Diagnostic and Interventional Neuroradiology, University Hospital Carl Gustav Carus, Dresden, Germany; 5 Radiologie Bamberg, Bamberg, Germany; 6 Institute of Neuroradiology, TUM University Hospital, Munich, Germany

**Keywords:** McDonald criteria to diagnose MS (2024 revisions), Dissemination in space and time, Central vein sign, Paramagnetic rim lesions, Susceptibility-weighted imaging

## Abstract

The recently published 2024 revisions of the McDonald criteria unified and expanded the diagnosis of MS towards a “biological diagnosis” and incorporated new MR imaging (bio)markers. Most prominently, they now incorporate imaging of the optic nerve as the fifth topography for dissemination in space (DIS), as well as the central vein sign (CVS) and paramagnetic rim lesions (PRLs), to facilitate an earlier diagnosis of MS. In addition, certain new MRI measures, such as slowly expanding lesions (SELs) and particularly PRLs, are increasingly used in clinical trials and may enter routine clinical practice in the near future. These developments, along with the 2024 MAGNIMS-NAIMS-CMSC consensus recommendations, suggest a different role for MRI in managing patients with MS and expansion of the MRI acquisition protocols, reading, and interpretation. Here, an imaging expert panel of the German Society of Neuroradiology (DGNR) established consensus recommendations on the role of MRI in the management of patients with suspected or definite MS, endorsed by the German Society of Neurology (DGN). These recommendations incorporate these new developments into the context of the German healthcare system. We suggest minor modifications to the acquisition protocol outlined in the 2024 McDonald criteria and the MAGNIMS-NAIMS-CMSC consensus recommendations. For patients with inconclusive imaging findings and in specific diagnostic situations, we recommend changes to MRI acquisition protocols that incorporate susceptibility-weighted imaging (SWI) sequences to demonstrate CVS and PRLs, as well as optic nerve imaging. Additionally, we provide recommendations on the use of MRI for treatment monitoring, including progressive MS patients.

**Supplementary Information:**

The online version of this article (https://doi.org/10.1007/s00062-026-01670-4) contains supplementary material, which is available to authorized users.

## Introduction

Multiple sclerosis (MS) is the most frequent chronic inflammatory disease of the central nervous system (CNS) in young adults [1]. In addition to the clinical presentation, neurological examination, and cerebrospinal fluid (CSF) analysis, magnetic resonance imaging (MRI) of the brain and spinal cord is essential for diagnosing MS. With the most recent revisions to the McDonald criteria, this role is expanding further [2, 3]. In addition, brain and spinal cord MRIs play a crucial role in prognostic classification of individual patients at the time of diagnosis, as well as in treatment monitoring and assessing treatment efficacy [4]. In addition to conventional MRI, i.e., markers of T2-weighted (T2w)-hyperintense inflammatory lesions in specific anatomic locations as well as contrast-enhancing lesions, new MRI markers have been proposed for diagnostic and treatment monitoring purposes, namely optic nerve involvement and particular lesion characteristics based on susceptibility-weighted imaging (SWI), i.e., the central vein sign (CVS) and paramagnetic rim lesions (PRLs) [5–7].

Recently, the 2024 revisions of the McDonald criteria have incorporated some of these additional imaging markers, such as optic nerve (ON) involvement as a fifth anatomic topography for demonstrating dissemination in space (DIS) and the presence of the CVS and PRLs in patients with typical symptoms and typical lesion(s) in at least one topography to diagnose MS [2]. First validation studies suggest earlier MS diagnosis than under previous diagnostic criteria, allowing an MS diagnosis even in patients without clinical symptoms or typical MRI findings, for example, when CVS positivity is demonstrated in radiologically isolated syndrome (RIS) and non-specific clinical presentations [8]. The accompanying 2024 MAGNIMS-NAIMS-CMSC consensus recommendations suggest expanding the core MR acquisition protocol to routinely include imaging techniques that facilitate the assessment of CVS and PRLs [3]. However, there is currently insufficient clinical validation of whether incorporating the CVS and PRLs into routine clinical settings will substantially improve the diagnostic process in patients with suspected MS. Moreover, the stringent application of these criteria in the clinical routine will significantly increase the utilization of required resources, including MR acquisition time (i.e., for ON-MRI and SWI) and image analysis and interpretation (i.e., for the accurate assessment of PRLs and CVS). In many countries, this may not be feasible on a regular basis in everyday clinical practice and will not be reimbursed appropriately. Furthermore, the consistent and correct implementation of these MRI markers requires a high degree of expertise and training in image acquisition and analysis, and the lack of dedicated expertise may rather lead to inaccurate image interpretation with considerable consequences for diagnosis and clinical management.

In this expert panel consensus paper, endorsed by the German Societies of Neuroradiology (DGNR) and Neurology (DGN), we present clinical practice guidelines for the use of MRI in the diagnosis and monitoring of MS patients, while acknowledging and accounting for the resources of the German healthcare system, its current capacities and reimbursement policies for radiological services.

## Methods

### Consortium Composition and Governance

The consortium comprised seven board-certified neuroradiologists and neuroimaging experts in the field of neuroinflammatory diseases from six institutions across Germany, four of which were affiliated with academic medical centers and two with private medical centers specialized in MS imaging. The consortium was founded on the initiative of individuals, after which additional members were invited to ensure that clinicians with direct experience in diagnostic MRI for MS, including subspecialty expertise in optic nerve, spinal cord, and advanced lesion imaging, were included. MPW served as convenor and manuscript coordinator; topic leads were appointed for each predefined area. No industry or other funding organization provided funding for the project, excluding any influence on topic selection, meeting conduct, or recommendation content by third parties.

### Meeting Format and Timeline

Between November 2024 and December 2025, the group convened eight virtual meetings. Meetings were scheduled to allow pre-circulation of materials and to maximise participation. The first five meetings addressed predefined technical topics: conventional brain MRI, optic nerve, PRL, CVS, and spinal cord imaging. A sixth meeting addressed broader methodological and implementation issues; two additional meetings were reserved for final consensus and manuscript drafting.

### Preparation of Evidence Summaries

For each of the five topic meetings, a topic lead conducted a targeted literature review and prepared a 20–30-minute evidence summary slide presentation. Literature searches prioritized peer-reviewed studies, systematic reviews, and recent guideline statements relevant to the 2024 McDonald criteria and imaging biomarkers. When evidence was limited or heterogeneous, topic leads highlighted areas of uncertainty and practical considerations drawn from local experience and expertise.

### Discussion and Development of Recommendations

Each topical meeting followed a structured agenda: (1) brief presentation of the evidence by the topic lead; (2) open discussion of methodological issues, technical parameters, and clinical implications; (3) proposal of draft recommendation statements. Discussions focused on reproducibility, technical feasibility in routine clinical practice, and compatibility with the 2024 McDonald criteria. Technical parameters (e.g., sequences, field strength, slice thickness) were specified where evidence supported particular recommendations; otherwise, pragmatic options were discussed and agreed upon.

### Consensus Process

Recommendations were refined in real time during meetings and then circulated as a revised draft to all members for review. We used a modified Delphi-style approach to achieve consensus without formal anonymity: after initial in-meeting agreement, members indicated their level of agreement with each recommendation. A recommendation was considered accepted when a majority of the panel members agreed. Recommendations that did not meet the acceptance threshold were revised by the topic lead to address the identified concerns and subsequently recirculated.

### Integration and Manuscript Drafting

Outcomes from the five topical meetings were synthesised into a single set of recommendations during dedicated integration meetings and extensive email discussions. The integration phase aligned technical recommendations with the 2024 McDonald criteria, clarified implementation pathways for routine practice, and generated graded implementation statements (mandatory, recommended, optional) based on the strength of evidence and feasibility. The manuscript was drafted by a writing subgroup, circulated for iterative comment, and approved by all consortium members before submission.

### Reporting Standards

This methodological process follows established approaches used by multisociety imaging consortia and guideline panels for producing pragmatic, evidence-informed technical recommendations. We report both consensus recommendations and areas of uncertainty to inform future research and local adoption.

## Results

### Use of MRI for MS Diagnosis According to the 2024 McDonald Diagnostic Criteria

In general, the use and interpretation of MRI in patients with suspected MS should be performed in conjunction with assessment of the clinical presentation and fluid biomarkers, such as cerebrospinal fluid (CSF) specific oligoclonal bands and/or the kappa-free light chain index [9]. Standardization of MRI acquisition parameters, including scanner hardware (e.g., MRI systems and coils) and software, patient repositioning, and parameters related to pulse sequences and spatial resolution, is a prerequisite for reproducible assessment of clinically relevant diagnostic as well as prognostic imaging markers and for consistent longitudinal follow-up in the evaluation of disease activity and treatment response [3, 10–12].

#### Image Acquisition Protocols

According to previous and current guidelines, contrast-enhanced brain MRI and whole spinal cord MRI are strongly recommended at the initial diagnostic workup [3, 10–12]. Detailed information on the acquisition protocols is given in Tables 1 and 2.

**Table 1:** MR imaging acquisition protocols

	Brain	Spinal cord	Optic nerve
**a. General recommendations**			
Field strength	≥ 1.5 T 3 T recommended	≥ 1.5 T	≥ 1.5 T
Image acquisition	3D or 2D 3D for T2w FLAIR^1^	2D or 3D (for 3D, ensure sufficient lesion contrast)	2D or 3D
Spatial resolution	2D: slice thickness ≤ 3 mm, no gap, in-plane resolution ≤ 1 mm × 1 mm 3D: ≤ 1 mm isotropic voxel size	2D: sagittal slice thickness ≤ 3 mm, no gap axial slice thickness ≤ 3 mm, no gap	2D: slice thickness ≤ 2–3 mm, no gap 3D: ≤ 1 mm isotropic voxel size
Scan orientation	Axial (subcallosal plane or ACPC line, resp.) and sagittal	Sagittal and axial	Coronal and axial
Coverage	Whole brain 3D should include the upper 3 segments of the cervical spinal cord	Whole spinal cord, including the conus	Whole optic nerve and optic chiasm
**b. Sequence recommendations**			
*Diagnosis*			
Core protocol	> 3D *or* axial and sagittal 2D T2w FLAIR^1^ > Axial 2D T2w TSE > 3D *or* axial 2D T1w + Gd^2^	> Sagittal 2D T2w TSE *with one additional complementary sequence, either*: Sagittal PD or T2w STIR > Axial 2D T2w > Sagittal 2D T1w + Gd^2^	> Coronal 2D (or 3D) T2w fat-saturated/STIR > Axial 2D (or 3D) T2w fat-saturated/STIR
Additional recommended sequences	> 3D SWI^4^	–	> Coronal 2D T1w fat-saturated + Gd^2^
Optional sequences	> Diffusion-weighted imaging (DWI) > 3D (heavily) T1w without Gd^5^ > 3D T2w DIR	> Axial 2D T1w + Gd^2^ > 3D T1w PSIR *or* 3D MP[2]RAGE *or* 3D T2w DIR *or* SPACE	> Axial 2D T1w fat-saturated + Gd^2^ > 3D T2w DIR
*Monitoring*			
Core protocol	> 3D *or* axial and sagittal 2D T2w FLAIR^1^ > Axial 2D T2w (T)SE/(F)SE^7^	> Sagittal 2D T2w TSE *with one additional complementary T2w sequence, either*: Sagittal PD or T2w STIR^6^ > Axial T2w^6^	Optic nerve imaging is not recommended for monitoring purposes
Additional recommended sequences	> 3D SWI	–	Optic nerve imaging is not recommended for monitoring purposes
Optional sequences	> Axial 2D T1w + Gd^2,3^ > Diffusion-weighted imaging (DWI) > 3D (heavily) T1w without Gd^5^ > 3D T2w DIR	> 3D T1w PSIR *or* 3D MP[2]RAGE *or* 3D T2w DIR *or* SPACE	Optic nerve imaging is not recommended for monitoring purposes

*D* dimensional, *T* tesla, *FLAIR* fluid attenuated inversion recovery, *STIR* short tau inversion recovery, *PSIR* phase sensitive inversion recovery, *DIR* double inversion recovery, *SPACE* Sampling Perfection with Application optimized Contrasts using different flip-angle Evolutions

^1^ 3D T2w FLAIR imaging is strongly recommended. Fat suppression is optional, but recommended

^2^ Standard dose of 0.1 mmol/kg body weight for established macrocyclic GBCA. For next-generation GBCAs, the efficacy of potentially lower doses remains to be prospectively validated. Minimum delay before T1w image acquisition of 5–7 min

^3^ Routine acquisition of contrast-enhanced images at regular follow-up is not recommended

^4^ In cases outlined in the section “Diagnostic algorithm”, SWI is strongly recommended to be acquired

^5^ Recommended for centers that want to include atrophy measurements for clinical decision-making and research purposes

^6^ Spinal cord MRI not recommended for routine monitoring purposes (Table 2)

^7^ In challenging cases or when 2D T2w FLAIR sequences or 3D T2w FLAIR sequences of insufficient quality are acquired, axial T2w (T)SE/(F)SE is recommended as part of the core monitoring protocol

**Table 2:** Recommendations for the use of MRI for establishing MS diagnosis

*Brain protocol*	3 T recommended, 1.5 T sufficient. 7 T is not recommended for the clinical routine setting
	Core sequences: 3D T2w FLAIR, axial 2D T2w, and 3D or axial 2D T1w with Gd (Table 1; Fig. 1)
	SWI and optic nerve imaging are recommended in certain situations (Figs. 4, 5 and 6)
*Spinal cord protocol*	1.5 or 3 T (higher magnetic fields not generally superior)
	Core sequences: Sagittal 2D T2w TSE with either PD or T2w STIR, axial T2w TSE, sagittal T1w with Gd, covering the entire spinal cord (Table 1; Fig. 1)
*Contrast media*	Exclusively single-dose (0.1 mmol/kg body weight) macrocyclic Gd-based contrast agents (GBCA) should be administered
	A minimum delay of 5 min between contrast administration is needed
*Additional and optional MRI techniques*	DWI cannot replace Gd as a marker for active inflammation
	Brain volumetric measurements
	Other advanced MRI techniques (e.g., DTI, fMRI, MTR)
	3D T2w DIR and/or T1w PSIR for cortical lesions
	3D MP[2]RAGE and T1w PSIR for spinal cord imaging
*Follow-up imaging to establish MS diagnosis*	Brain MRI without contrast is recommended 4–6 months after the initial MRI
	Routine spinal cord MRI follow-up is not recommended
	Identical image acquisition (i.e., standardized repositioning, field strength, pulse sequences, spatial resolution) is strongly recommended
*Image interpretation*	Standardized image interpretation and reporting are crucial
	Knowledge of lesion types and their classification is crucial, and red flags should be recognized
	Standard measures (T2 lesion count, T2 lesion volume if available, Gd lesion count if Gd was administered) are recommended
	Separate identification of cortical lesions (together with juxtacortical lesions) based on standard images, e.g., T2w FLAIR (T2w DIR/T1w PSIR sequences optional)

#### General MR Recommendations and MR Core Sequences

The use and interpretation of MRI in the context of MS diagnosis is still based on the differential diagnostic “*concept of no better explanation,*” and lesion interpretation and classification should be performed in accordance with dedicated guidelines, with relevant MS differential diagnoses considered [13–15].

#### Magnetic Field Strength

Higher magnetic field strengths, operating at 3 T, provide higher signal-to-noise ratios (SNR) and improved spatial resolution, thereby increasing lesion detection sensitivity, particularly for small lesions. However, the actual impact on classification according to diagnostic criteria remains low [16, 17]. For brain MRI in patients with suspected or established MS, a magnetic field strength of 3 T is recommended when available, but not mandatory. State-of-the-art 1.5 T MR systems, using appropriate coils and optimized acquisition parameters, can yield high-quality imaging comparable to that of 3 T acquisitions.

The use of magnetic field strengths beyond 3 T (e.g., 7 T) is not recommended for routine clinical practice, mainly for two reasons: (1) 7 T systems are not yet widely available, and (2) image interpretation remains challenging due to the effects of ultra-high field strength on tissue relaxation times and image contrast, which have not yet been fully investigated, potentially increasing the risk of misinterpretation [18]. Other reasons are increased specific absorption rates (SAR) and their limits along with patient comfort (mainly related to increased examination times and sensations of heating).

#### Brain

##### Core Sequences

**Fig. 1:**
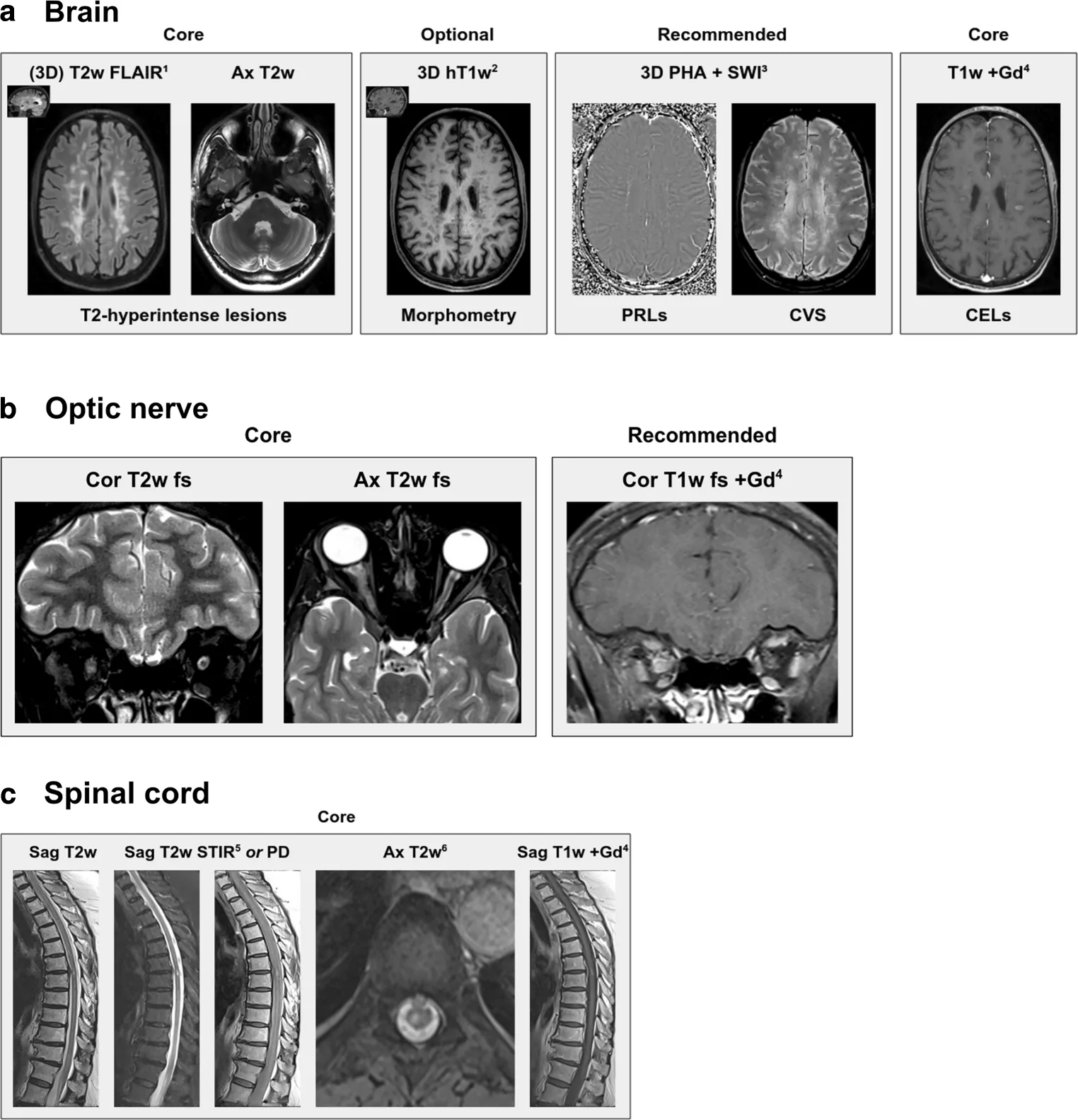
Overview of protocol recommendations for brain (**a**), optic nerve (**b**) and spinal cord (**c**). ^1^ 3D T2w FLAIR imaging is strongly recommended. Fat suppression is optional, but recommended. ^2^ Heavily 3D T1w is recommended for centers that want to include atrophy measurements for research purposes. ^3^ In cases outlined in the section “Diagnostic algorithm”, SWI is strongly recommended to be acquired. ^4^ Standard dose of 0.1 mmol/kg body weight for established macrocyclic GBCA. For next-generation GBCAs, the efficacy of potentially lower doses remains to be prospectively validated. Minimum delay before T1w image acquisition of 5–7 min. ^5^ T2w STIR should not be acquired after GBCA application. ^6^ Cover the entire spinal cord.

The core MRI acquisition protocol consists of T2w FLAIR imaging (preferably as a 3D acquisition with ≤ 1 mm isotropic resolution and multiplanar reformats) for lesion identification, as well as axial T2w fast/turbo spin-echo imaging (slice thickness ≤ 3 mm, no interslice gap) particularly to detect or confirm posterior fossa and deep grey matter lesions (Fig. 1a). Using 3D acquisition techniques instead of 2D acquisition, particularly for T2w FLAIR imaging, improves lesion detection and interpretability and is therefore recommended [19, 20].

In the diagnostic setting, the core protocol should also routinely include contrast-enhanced T1-weighted (T1w) imaging to determine if dissemination in time (DIT) criteria are fulfilled, by demonstrating the simultaneous presence of both enhancing and non-enhancing lesions, and to rule out potential differential diagnoses [3]. Spin-echo (SE) and turbo/fast spin-echo (TSE/FSE) sequences, as well as simple gradient-recalled echo (GRE) sequences, are most suitable for detecting contrast-enhancing MS lesions and provide excellent lesion conspicuity [21, 22]. Conversely, heavily T1w sequences (e.g., MPRAGE (magnetization-prepared rapid gradient-echo) or PSIR (phase-sensitive inversion recovery)) reduce the detectability of enhancing lesions because of elevated background white matter signal [13]. Besides assessing DIT, contrast-enhanced imaging plays an important role for prognostic purposes and to exclude important differential diagnoses, such as neurosarcoidosis or neuroinfectious diseases, also in patients without or with inconclusive T2-hyperintense lesions. A minimum delay of 5 min between contrast administration and post-contrast T1w imaging is advised to facilitate the sufficient detection of enhancing lesions. In alignment with the ESMRMB-GREC and ESNR Multiple Sclerosis Working Group [23], exclusively single-dose macrocyclic gadolinium-based contrast agents (GBCAs) should be administered. For established GBCAs, the recommended dose is 0.1 mmol/kg of body weight. For next-generation GBCAs, the efficacy of potentially lower doses remains to be prospectively validated. Importantly, contrast-enhanced T1-weighted imaging is generally not required for routine disease monitoring [12, 23, 24].

#### Non-Core Sequences

The 2024 MAGNIMS–CMSC–NAIMS consensus guidelines recommend the inclusion of a 3D T2*-weighted (T2*w) gradient-recalled-echo (GRE) sequence (e.g., echo-planar imaging (EPI) or other optimized susceptibility-weighted imaging (SWI) sequences) in the core protocol [3]. This sequence is not included in our routine recommendations for various reasons. SWI and the assessment of CVS and PRLs are required only in very specific clinical scenarios (see section “*Imaging susceptibility*” below). Furthermore, there is currently no clearly demonstrated benefit of using these sequences for treatment monitoring purposes yet (see section “*MRI measures for the assessment of inflammatory disease activity*” below). Therefore, we regard SWI and related techniques as adjunctive rather than core MRI sequences.

Pre-contrast high-resolution 3D heavily T1w imaging is, in accordance with the 2021 and 2024 MAGNIMS–CMSC–NAIMS consensus guidelines, also not yet recommended in the general core MRI protocol (Table 1). However, such sequences, particularly when heavily T1w (e.g., MPRAGE) and recorded at isotropic high spatial resolution, can provide baseline information for centers using morphometric data for additional clinical or research purposes or for volumetric monitoring. Therefore, these sequences are recommended for centers seeking to include atrophy measurements for research. Additionally, certain sequences, such as T1w PSIR and T2w Double Inversion Recovery (DIR), can improve the detection and classification of cortical lesions [25–27]. (Juxta-)cortical lesions are among the five topographies that demonstrate DIS according to the 2024 revisions of the McDonald criteria and are relevant for the prognostic classification of an individual patient [28]. However, standardized, high-quality acquisition, as well as the interpretation of cortical lesions, remains challenging, time-consuming, and exhibits high inter-rater variability, requiring expertise and experience [29]. Therefore, these sequences are not yet recommended for general clinical practice.

Diffusion-weighted imaging (DWI) is frequently included in routine MRI protocols for miscellaneous clinical conditions, particularly for the detection of (sub)acute brain infarction. In patients with CNS inflammatory diseases, acute demyelinating lesions can present with high signal intensity on DWI and a corresponding low apparent diffusion coefficient (ADC) [30]. It has been demonstrated that this diffusion restriction can occur before subsequent blood-brain barrier disruption, leading to contrast enhancement [30, 31]. However, this observation has not yet been validated in larger cohorts. Therefore, according to previous guidelines, the use of DWI as a marker for acute or active inflammation remains not recommended and cannot be considered as a surrogate marker of acute demyelination. Consistent with previous guidelines, other advanced and quantitative MRI techniques, such as diffusion tensor imaging (DTI) and functional MRI (fMRI) [32], are also not recommended for clinical routine use [3, 10–12].

#### Imaging the Optic Nerve (ON)

Optic neuritis is a frequent first presentation of MS but is also seen in important differential diagnoses, such as Neuromyelitis Optica Spectrum Disorders (NMOSD) and Myelin Oligodendrocyte Glycoprotein Antibody-associated Disease (MOGAD) [33, 34]. The 2024 revisions of the McDonald criteria include the optic nerve (ON) as a fifth anatomic location for the demonstration of DIS, emphasizing the increased relevance of ON MR imaging for the diagnosis of MS, alongside visual evoked potentials (VEP) and optical coherence tomography (OCT) [2, 35]. ON-MRI acquisition protocols include coronal and axial fat-suppressed T2w such as T2w STIR (Short Tau Inversion Recovery) and fat-suppressed Gd-enhanced T1w sequences (Table 1; [34]). The location and extension of ON lesions, as well as their contrast enhancement, can help differentiate MS from ON involvement in important differential diagnoses, such as NMOSD and MOGAD [36]. A multicenter trial showed a slight increase in patients fulfilling DIS when ON involvement on ON-MRI was considered, a finding that could not be reproduced by others [6, 37]. Recent MAGNIMS recommendations advise the use of ON-MRI regularly in all patients with suspected MS [38]. Based on the currently available data and given that including full ON-MRI in the core imaging protocol would increase in acquisition time significantly, we recommend to use ON-MRI exclusively in patients with either inconclusive imaging findings that do not as such already fulfill McDonald diagnostic criteria, based on their brain and spinal cord MRI, and in whom VEP and OCT are unavailable, inconclusive or negative, and in patients with clinical presentations atypical for ON (Fig. 1b). We recommend that centers incorporating ON-MRI into routine protocols use coronal fat-saturated T2w imaging as the main screening sequence for optic nerve lesions. Fat-suppression methods that simultaneously provide unsuppressed images from the same acquisition (such as DIXON) can be useful in certain differential diagnoses (e.g., optic nerve sheet meningiomas) based on casuistic experience. To meet scan time constraints, both optic nerves may be imaged simultaneously (although this inevitably entails slightly oblique, non-perpendicular slicing along their course, which, at least in theory, is not optimal). Note that the average intraorbital height (2.3 mm, without sheets) and corresponding cross-sectional area (4.2 mm^2^) of the ONs are small [39]. Therefore, in-plane resolution of ≤ 1 × 1 mm and 2–3 mm-thick slices with no gap should be considered a minimum requirement for imaging optic neuritis. It is currently not clear how well 3D T2w FLAIR or T2w DIR (double-inversion recovery; suggested by the 2024 MAGNIMS recommendations as “additional”) sequences with full brain and ON coverage generally perform to detect ON lesions. Based on the available evidence, our panel included T2w DIR for ON-MRI as “optional” [40].

#### Imaging Susceptibility

The central vein sign (CVS) and paramagnetic rim lesions (PRLs) are new MRI biomarkers incorporated into the 2024 revision of the McDonald criteria to diagnose MS [2]. CVS corresponds to a tubular MRI signal hypointensity within a white matter lesion in primarily susceptibility-sensitive imaging sequences that depicts a transmedullary vein in the center of the lesion [41]. CVS has been proposed as an imaging marker to help distinguish MS from other central nervous system diseases that may mimic MS, particularly ischemic small-vessel disease [42–44]. Previous studies have demonstrated that a high proportion of perivenular lesions, typically more than 50% of all visible lesions, confers high specificity for MS [45, 46]. However, determining such lesion proportions is time-consuming and impractical in routine clinical settings. Therefore, the introduction of the “select‑6 rule,” defined as the presence of six or more lesions exhibiting a central vein sign, represents a pragmatic, clinically feasible alternative with very high specificity [7, 47]. The “select‑6 rule” has been incorporated into the 2024 revisions of the McDonald criteria for patients with more than 9 lesions [2, 7]. For fewer than 10 lesions, the majority of lesions should exhibit CVS (“majority rule”).

PRLs (synonym: phase rim lesions) are a specific type of inflammatory focal brain lesion detectable by susceptibility-sensitive MRI and are considered a specific biomarker of chronic active inflammation, particularly in MS [48–50]. Based on neuropathology studies, these lesions are characterized by a rim of iron-containing immune cells that equip PRLs with their paramagnetic properties [51, 52]. The presence of a single PRL has been shown to be highly specific to MS and can support diagnosis and distinction from other inflammatory diseases of the CNS [53–56].

#### SWI Acquisition

Both CVS and PRLs should be assessed using susceptibility-sensitive MRI pulse sequences, preferably using a single acquisition. SWI is the predominant technique for this purpose. Its distinctive feature is that it uses phase information to distinguish between paramagnetic substances (e.g., deoxygenated haemoglobin, ferritin, and hemosiderin) and diamagnetic compounds (e.g., calcifications), which is not possible with magnitude-only information.

In general, any T2*w GRE sequence, e.g., EPI, FISP, FLASH, RUFIS, VIBE etc. (see Table 3), can be used for SWI. There is considerable terminological confusion regarding SWI, as there are various generic trade names from different vendors for it (*Siemens: SWI, Philips: SWIp=SWI-phase/formerly VenoBOLD, GE: SWAN=T2-Star Weighted Angiography and Looping Star RUFIS=3D radial Rotating Ultra-Fast Imaging Sequence, Fuji: BSI=Blood Sensitive Imaging, Canon: FSBB=Flow Sensitive Black Blood*).

**Table 3:** Technical Recommendation for SWI Acquisition

**Magnetic field strength:** ≥ 1.5 T, 3 T strongly recommended
**Pulse sequence: **any phase-sensitive GRE-SWI (e.g., EPI, FISP, FLASH, RUFIS, VIBE, etc.), minimized for geometric distortions (e.g., readout segmentations in EPI), 3D acquisition recommended
**Echo time (TE): **depending on pulse sequence and field strength used (≥ 20 ms for FLASH at 3 T / ≥ 35 ms for EPI and at 1.5 T, or across multiple TEs)
**Flip angle (FA): **approx. 5°–10° to ensure that MAG is sufficiently T2*-weighted. The disadvantage is SNR loss, especially at 1.5 T. Alternatively, the TE/TR ratio can be increased
**Spatial resolution: **≤ 0.8 × 0.8 mm in-plane minimally recommended (≤ 0.5 × 0.5 mm without interpolation optimal if achievable), ≤ 3 mm through-plane (≤ 2 mm if achievable), no gap; *High spatial resolution, preferably isotropic, is a priori more important for CVS/PRL detection than the pulse sequence type used *(assuming sufficient image quality, esp. SNR/CNR)
**Coverage: **entire supra- and infratentorial brain (*whole brain*)
**Angulation:** strictly axial to the magnet coordinate system
**Acquisition time (TA): **typically between 2.5 and 6 min (dependent on sequence type, spatial resolution, and acceleration techniques used)
**Image reconstruction: **separate PHA and MAG/PHA-combined SWI output reconstructed, MinIP is dispensable for MS diagnostic and monitoring purposes
**Gadolinium administration: **optional and relevant for CVS only, if applied: macrocyclic Gd, 0.1 mmol/kg BW, no (or minimized) delay with respect to SWI acquisition

*BW* body weight, *CNR* contrast-to-noise ratio, *CVS* central vein sign, *Gd* Gadolinium, *EPI* echo planar imaging, *GRE* gradient echo, *FISP* fast imaging with steady-state precession, *FLASH* fast low angle shot, *MAG* magnitude reconstruction (of SWI sequence), *MinIP* minimum intensity projection (of MAG/PHA-combined SWI), *PRL* paramagnetic rim lesion, *PHA* processed (= unwrapped, high-pass filtered and masked) phase reconstruction (of SWI sequence), *RUFIS* rotating ultra-fast imaging sequence, *SNR* signal-to-noise ratio, *SWI* susceptibility-weighted imaging, *VIBE* volumetric interpolated brain examination

3D T2*w GRE EPI has been suggested as optimal for CVS detection [2]. However, 3D T2*w GRE EPI with magnitude and phase reconstruction is actually a SWI sequence, and the diagnostic superiority of one method over others for detecting CVS or PRLs has not been conclusively demonstrated [57]. Furthermore, GRE EPI suffers from specific artefacts (geometric EPI distortions, ghosting, GRE-related signal dropouts, off-resonance field, phase and susceptibility irregularities), and is generally not optimal in areas close to the pneumatized skull base, for example.

SWI acquisitions provide up to four different outputs: (1) magnitude reconstructions (MAG) with variable or mixed T1/T2* weighting, (2) unwrapped, high-pass filtered, and usually masked phase reconstructions (PHA), (3) the actual SWI itself which is magnitude and phase combined by further postprocessing (i.e., MAG multiplied several times by PHA) and therefore a “*mixed contrast*”, and (4) minimum intensity projections (MinIP) of the SWI, which are intended in particular for MR venographic overviews. Due to the involved projection across predefined, usually axial slabs and the corresponding loss of spatial discrimination, MinIPs are not suitable for CVS and PRL assessment. Note that the direction (“*handedness*”) of phase shift encoding is manufacturer-dependent, i.e., deoxyhemoglobin in the cerebral veins of the CVS and paramagnetic compounds of the PRLs may appear light or dark in the processed phase outputs, depending on manufacturer conventions.

Basically, MAG can be more T1- or T2(*)-weighted. Shortening the repetition time (TR) and increasing the flip angle (FA; to approx. 15° or more) increases T1 weighting, while lengthening the echo time (TE) and reducing the FA (to approx. 5–10°) results in more predominant T2(*) weighting. Such weighting differences subsequently affect the MAG/PHA-combined SWI. If Gd is administered before acquisition, any focal lesion enhancement or enhancement in the transmedullary veins on predominantly T1w MAG may attenuate the susceptibility-related hypointensity in the reconstructed SWI. PHA, on the other hand, is essentially insensitive to T1-shortening caused by GBCA. However, T2 lesion hyperintensity in T2(*)w MAG may attenuate PHA information in the combined SWI. These effects are relevant for CVS and PRL detection [58].

#### Image Analysis of Susceptibility-Weighted Imaging

In line with the SWI MRI acquisition protocol guidelines given in Table 3, we recommend using the SWI sequence most readily available on the local MR system and optimizing it for concurrent detection of both, CVS and PRLs. Until proven otherwise, it is unlikely that 3D T2*w GRE segmented EPI will outperform other SWIs in CVS and PRL detection, especially when SNR and spatial resolution are matched. However, it is indeed of utmost importance to optimize the spatial resolution and, in particular, the slice thinness of the respective SWI to achieve isotropic or near-isotropic resolution—otherwise, detecting and confirming PRLs and, in particular, CVS, on a perpendicular plane or on two consecutive slices can be challenging or even fail simply due to limited and anisotropic spatial resolution [57]. For MS follow-ups, we urge to use the same sequence specifications for repeated diagnostic imaging to reduce variability and avoid misinterpretations. The individual identification and exclusion criteria for CVS and PRLs can be found in Tables 4 and 5, general recommendations for SWI evaluation in Table 6. Note that these are less rigorous evidence- and rather convention-based criteria. Therefore, they must be critically considered and applied. For example, the CVS lesion size exclusion criterion (less than 3 mm in all planes) crucially depends on the underlying spatial MRI resolution, how planes are reconstructed, and how diameters are measured. The same holds true for the inclusion criterion for PRL, which requires the PRL to be identifiable on two consecutive slices of 2D acquisitions or in two perpendicular planes in 3D acquisition. Here, it matters significantly whether 0.5 or 3 mm slices are reconstructed, both of which are endorsed by the 2024 MAGNIMS-NAIMS-CMSC consensus recommendations [3]. The corresponding variability and limitations must be acknowledged.

**Table 4:** Identification and exclusion criteria for CVS, slightly modified from [42]

**Identification**
The shape of the CVS appears as a thin hypointense line or dot within a T2 lesion
Detectable in at least two consecutive slices or two perpendicular planes, with at least one plane showing a thin line (provided that adequate 3D resolution has been achieved)
Has a diameter of less than 2 mm
Runs completely or partially through a T2 lesion
Is always roughly equidistant from the lesion margins, i.e., runs centrally through the lesion
Enters and exits the lesion only once, regardless of the shape of the lesion
**Exclusion**
The corresponding T2 lesion has a diameter of less than 3 mm in each plane
Located in confluent lesions, i.e., lesion conglomerates (*not countable*)
Multiple veins are present within one lesion (*not countable*)
Insufficient delineation of the corresponding T2 lesion, e.g., due to artifacts

*CVS* central vein sign

**Table 5:** Identification and exclusion criteria for PRLs, slightly modified from [50, 92]

**Identification**
The PRL has a distinguishable paramagnetic thin rim on the margin of a T2-hyperintense lesion
The paramagnetic rim completely encompasses at least 2/3 of the outer WM lesion margin, with juxtacortical and periventricular lesions exempt from this requirement (*half rims*)
Colocalization of the rim with the entire margin, or edge and part of the core of an underlying T2-hyperintense lesion. Alternatively, PRL cores may be more readily identified by the corresponding T1-hypointense lesion core in large, confluent T2 lesions
Identifiability of the rim on at least two consecutive slices (2D or 3D acquisition) or in two perpendicular planes (3D acquisition)
*Special requirement*: Proof of chronicity of the lesion for PRL
In Gd-enhanced MRI examinations, the PRL core, defined as a central lesion component that does not take up contrast agent, confirms the status of a chronic lesion
If no Gd was administered, the presence of the underlying T2 lesion in previous examinations must be verified to confirm chronicity
If neither Gd-enhanced MRI nor a previous examination is available, the PRL should be provisionally classified as “*possible*” and its chronicity “*confirmed*” by a follow-up examination 3–6 months later
**Exclusion**
If veins run along the edges of the lesion that resemble part of a paramagnetic rim
If the PRL core has a diameter of less than 3 mm in its largest dimension (*not countable*)
If the thickness of the paramagnetic margin is > 2 mm, and if changes in the susceptibility of the margin cannot be distinguished from those visible inside the lesion core
For lesions with limited assessability in anatomical regions with susceptibility artifacts

*PRL* paramagnetic rim lesion

**Table 6:** Recommendations for SWI evaluation

Use PHA to evaluate PRLs to distinguish the paramagnetic rim [58]
Use MAG/PHA combined SWI to evaluate CVS, but ensure that MAG is sufficiently T2*-weighted. Do not use MAG alone, as this will omit additional venographic benefits of PHA.
Consider Gd injection to improve CVS detection, but avoid injection delays with respect to SWI acquisition.
Ensure sufficient spatial resolution and SNR/CNR. Attempt to achieve high 3D resolution.

*CNR* contrast-to-noise ratio, *CVS* central vein sign, *Gd* Gadolinium, *MAG* magnitude reconstruction (of SWI sequence), *PRLs* paramagnetic rim lesions, *PHA* processed (= unwrapped, high-pass filtered and masked) phase reconstruction (of SWI sequence), *SNR* signal-to-noise ratio, *SWI* susceptibility-weighted imaging, *VIBE* volumetric interpolated brain examination

#### Central Vein Sign (CVS)

**Fig. 2:**
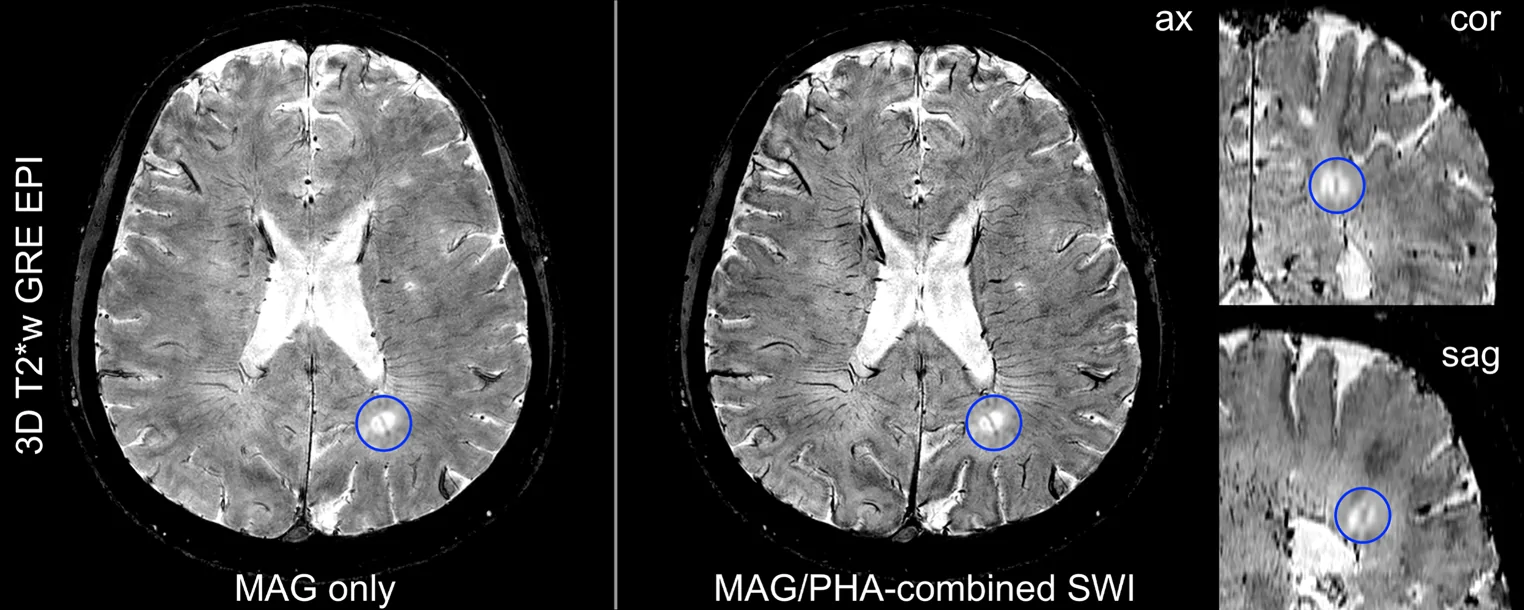
Detection of the central vein sign (*CVS*) as a new diagnostic MRI biomarker for MS by susceptibility-weighted imaging (*SWI*). CVS (blue circled), meeting the required identification/exclusion criteria (Table 4) and confirmed in three perpendicular planes by high-resolution 3D T2*w GRE EPI (0.44 × 0.44 × 1.00 mm^3^) in a patient with proven MS. Note that SWI improves CVS and, in general, transmedullary vein conspicuity compared to magnitude (*MAG*) only reconstructions because processed phase (*PHA*) is venographic. Therefore, CVS should be evaluated on MAG/PHA-combined SWI (Table 6). Note that 3D GRE EPI can also output processed PHA images for paramagnetic rim lesion (*PRL*) detection (cf. Fig. 3). We recommend using the same SWI for CVS and PRLs, resorting to the sequence most easily available on site and optimized for that purpose (cf. Table 3). Data courtesy of Thomas Illigen, Siemens Healthineers, images generated by AJB. Future product availability cannot be guaranteed.

CVS should be evaluated using MAG/PHA-combined SWI reconstructions according to the CVS inclusion and exclusion criteria based on the NAIMS Cooperative Consensus Statement on CVS (Fig. 2; [42]). The PHA component is strongly venographic and should never be omitted. Increasing the contrast-to-noise ratio (CNR) between hypointense central veins and surrounding MS lesions will inevitably improve the detectability of CVS. For this reason, T2*-weighting of MAG is beneficial for CVS sensitivity. If SNR is an issue, e.g., at 1.5 T, the alternative to reducing FA would be to increase TE (and TR, potentially along with acceleration techniques to speed up acquisitions). However, a direct comparison of the two approaches for T2*-weighting (i.e., reducing FA as suggested by the MAGNIMS-NAIMS-CMSC recommendations [3] *vs.* increasing TE and TR) is currently lacking.

In general, GBCAs are not needed at magnetic field strengths operating at 3 T. Whether or not it is attempted to further enhance the diagnostic utility of SWI for CVS detection by injecting GBCAs (Gadolinium-based contrast agents) should be decided at the institutional level. However, note that GBCAs are washed out of the veins some time after administration, and their application may therefore become futile with longer delays. Furthermore, venous Gd accumulation may even impair CVS detection in MAG/PHA combined SWI maps if MAG is considerably T1w. Therefore, when GBCAs are used, ensure that MAG is predominantly T2*w to avoid false-negative results (and to increase CNR between T2 lesion and CVS), and avoid delays or start SWI acquisition and GBCA injection simultaneously.

MinIPs are intended for venographic overview. However, MinIPs impair spatial discrimination by collapsing minimum intensities across a predefined slab of a number of slices. Therefore, MinIPs should not be used for CVS detection to avoid both false-negative as well as false-positive detections. If possible, automatic MinIPs reconstruction can be disabled for SWI protocols to diagnose and monitor MS.

#### Paramagnetic Rim Lesions (PRL)

**Fig. 3:**
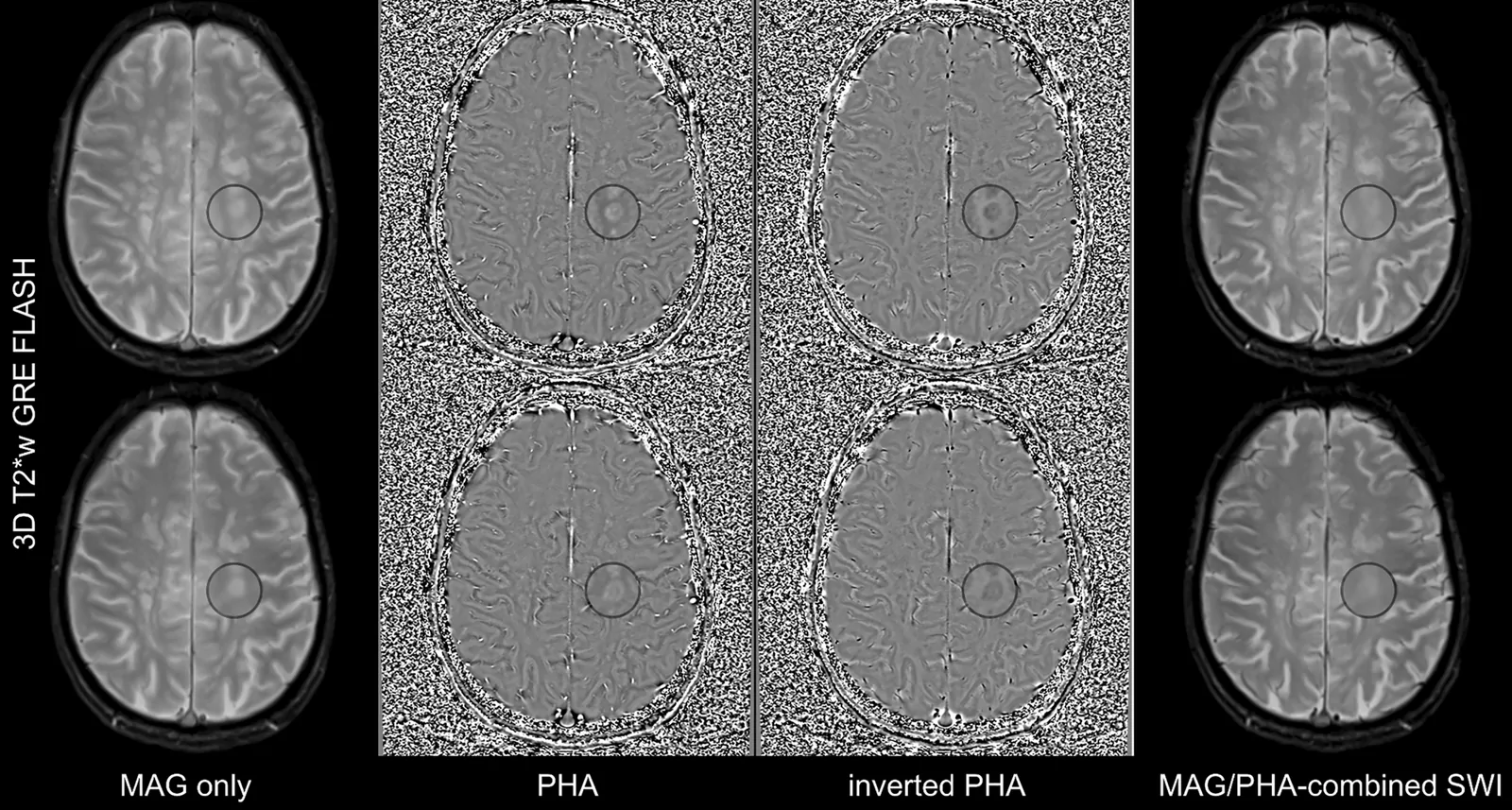
Detection of paramagnetic rim lesions (*PRLs*) as a new diagnostic and potentially prognostic MRI biomarker for MS by the processed phase component (*PHA*) of susceptibility-weighted imaging (*SWI*). PRL (black circled), meeting the required identification/exclusion criteria (Table 5) and confirmed on two consecutive slices by high-resolution 3D T2*w GRE FLASH (0.51 × 0.51 × 2.67 mm^3^) in a patient with proven MS. Note that T2-hyperintensity of MS lesions in T2*w magnitude (*MAG*) only reconstructions may null PRL-hypointensity in MAG/PHA-combined SWI [58]. Therefore, PRLs should be evaluated primarily on PHA (Table 6). White matter surrounding the PRL appears darkened on PHA (and brightened on inverted PHA), which is simply a Mach effect in the magnifier circle.

The paramagnetic rim of PRLs should be evaluated using PHA reconstructions primarily, as the mixed contrast of MAG/PHA-combined SWI can be deceptive in PRL identification (Fig. 3; [58]). This has three reasons: For one, T2 lesion hyperintensity of T2*w MAG counteracts PRL hypointensity in MAG/PHA-combined SWIs—an effect well-illustrated in a recent publication for intermediate MAG-weighting at FA = 10° [58]. Second, T1 lesion hypointensities can mimic paramagnetic deposits on MAG/PHA-combined SWI when MAG is predominantly T1w. Third, Gd-enhancement of MS lesions, such as peripheral ring enhancement, will attenuate or even null PRL hypointensities in MAG/PHA-combined SWIs when MAG is considerably T1w and GBACs are applied to improve CVS detectability. Therefore, generating appropriately preprocessed (i.e., unwrapped and high-pass filtered), high-quality PHA reconstructions as a separate output is essential for PRL detection [59]. Only in certain anatomical locations (such as the corticomedullary junction), where PRL detection on PHA may be impaired by interfering signals (e.g., from the cortex), it may be beneficial to increase T1-weighting of MAG and to evaluate PRLs on the MAG/PHA-combined SWI. As soon as a paramagnetic rim is detected on PHA, the presence of an underlying T2 lesion must be confirmed on T2*w MAG (which is naturally in register with PHA) or appropriately aligned or co-registered T2w FLAIR or T2w scans. This emphasizes that the diagnostic process of appropriate neuroradiological CVS and PRL identification is quite complex, laborious, and requires multiple steps.

The morphological spectrum of PRLs remains less well-defined than that of CVS, according to the NAIMS consensus definition [50]. For example, it remains debated to what degree the paramagnetic rim of PRLs can be interrupted or scattered, whether and how nodular or streak-shaped (i.e., not ring-like) paramagnetic lesions should be considered, how far the paramagnetic rim can extend into otherwise normal appearing white matter, and how thick or discrete it should be. The current identification and exclusion criteria for PRLs are outlined in Table 5. Even more than corresponding CVS criteria (cf. Table 4), these will require further refinements.

#### Spinal Cord

The high value of spinal cord MRI for diagnosing MS and for prognostic stratification at the time of diagnosis has been conclusively demonstrated [2, 4]. Spinal cord lesions are one of the five key anatomical regions for demonstrating DIS. In patients with ≥ 12 months of progression from onset, detection of ≥ two spinal cord lesions alone can replace DIS by substituting for an additional brain site [2]. Their presence is particularly important in older individuals or those with vascular risk factors, in whom at least one spinal cord lesion (along with CSF or CVS positivity) is strongly recommended to reduce false-positive MS diagnosis. In addition, spinal cord lesions also predict a higher and earlier risk of conversion from RIS to MS [60].

For MS diagnosis, we strongly recommend a contrast-enhanced MRI of the entire spinal cord at ≥ 1.5 T. The use of multi-array coils and prevertebral saturation blocks is advised. Following previous recommendations, the MR protocol should include a combination of sagittal T2w (turbo/fast) SE with moderately long echo times and either proton-density (PD) (turbo/fast) SE or T2w STIR [3, 61]. In addition, a sagittal Gd-enhanced T1w (turbo/fast) SE sequence should be performed (see Fig. 1c). T2w STIR must not be acquired after GBCA application, as T1-shortening may attenuate or even nullify T2 lesion conspicuity on T2w STIR [62, 63]. Therefore, if T2w STIR is chosen for sagittal spinal cord MRI, it should not be done so after Gd-enhanced brain imaging. A combination of sagittal PD and T2w STIR without T2-weighted (turbo/fast) spin-echo can be considered, but is not recommended, as PD and T2w STIR are prone to artefacts, potentially leading to inaccurate lesion detection and potential false-positives. Axial T2w (turbo/fast) SE or T2* (e.g., T2 medic) sequences are recommended at least in areas of a suspected lesion on the sagittal images and in areas with a high prevalence of MS lesions such as the cervical spinal cord to further improve the diagnostic certainty, particularly for differentiating MS from relevant differential diagnoses such as NMOSD or MOGAD [64, 65]. Full axial coverage of the entire spinal cord improves lesion detection and, if possible at the site, is recommended [66]. Note that we recommend ≤ 3 mm thin slices with no gap (Table 1), rather than ≤ 5 mm as specified by the 2024 MAGNIMS recommendations, to improve lesion detection, as spinal cord lesions can be rather small. Coverage of just the cervical segment of the spinal cord, minimally recommended by the 2024 MAGNIMS recommendations, may be insufficient because 10–20% of CIS patients present with spinal cord lesions at or below the level of C7 [67], and lower spinal cord involvement is more prevalent in MOGAD than in MS [68], i.e., important to detect for differential diagnosis. Sagittal T1w PSIR may serve as an alternative sequence to T2w STIR (or PD); however, its superiority, especially over T2w STIR in the clinical setting, remains not sufficiently investigated [69, 70].

Alternative 3D acquisition techniques such as 3D T1w sequences, e.g., magnetization prepared [2] rapid acquisition of gradient echoes (MP[2]RAGE), 3D T1w PSIR or 3D T2w DIR are increasingly being used for spinal cord imaging and possess a high sensitivity compared to conventional techniques such as T2w STIR and T2w (turbo/fast) SE, particularly in the cervical spinal cord [71–76]. Therefore, the 2024 MAGNIMS recommendations now include T1w PSIR and MP[2]RAGE in the set of complementary sagittal sequences (previously T2w STIR, T2w, and PD-weighted), from which two should be chosen for spinal cord MRI in the diagnostic work-up for MS. However, longer acquisition times and the clinical experience required for image acquisition and interpretation still limit their application in routine clinical practice. Additionally, image acquisition in the thoracic segment using these sequences remains a challenge. Therefore, our panel does not yet recommend such 3D sequences for clinical routine use.

### Diagnostic Algorithm

**Fig. 4:**
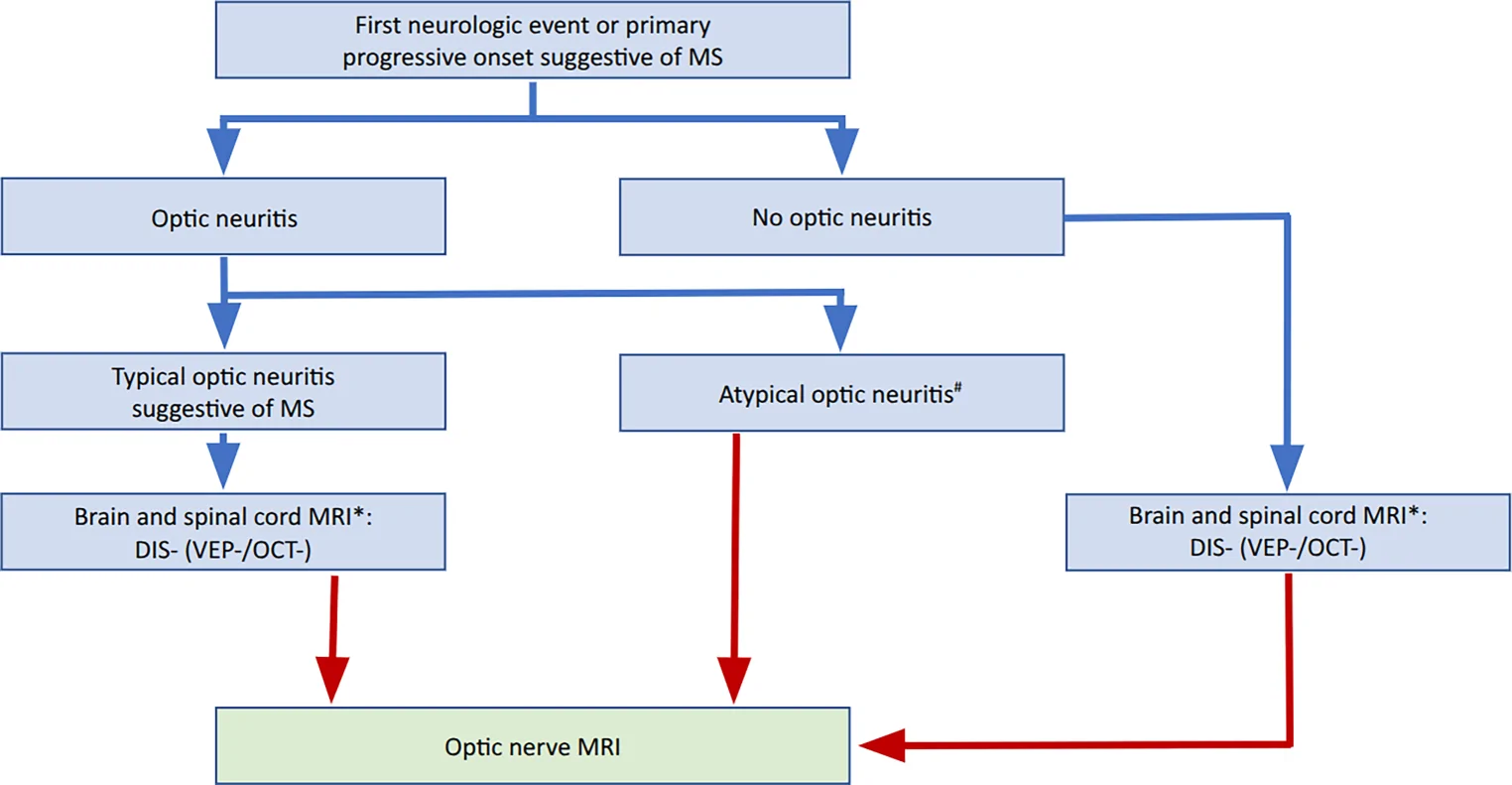
Flowchart on when optic nerve MRI is recommended to diagnose MS according to the 2024 revision of the McDonald criteria and 2024 MAGNIMS-CMSC-NAIMS consensus recommendations. *DIS* dissemination in space, *OCT* optical coherence tomography, *VEP* visual evoked potentials. * Compliant with 2024 MAGNIMS-NAIMS-CMSC consensus recommendations [3]. # Typical optic neuritis refers to presentations usually associated with MS-associated optic neuritis. Atypical presentations deviate from this and may clinically include severe visual loss, severe or no pain, lack of improvement, bilateral onset, etc. Non-MS-related presentations may also be suspected based on other features, e.g., MRI findings (longitudinally extensive lesions, lesion location, pattern of enhancement), OCT (degree of pRNFL swelling), and mismatch between the degree of degeneration & visual recovery

**Fig. 5:**
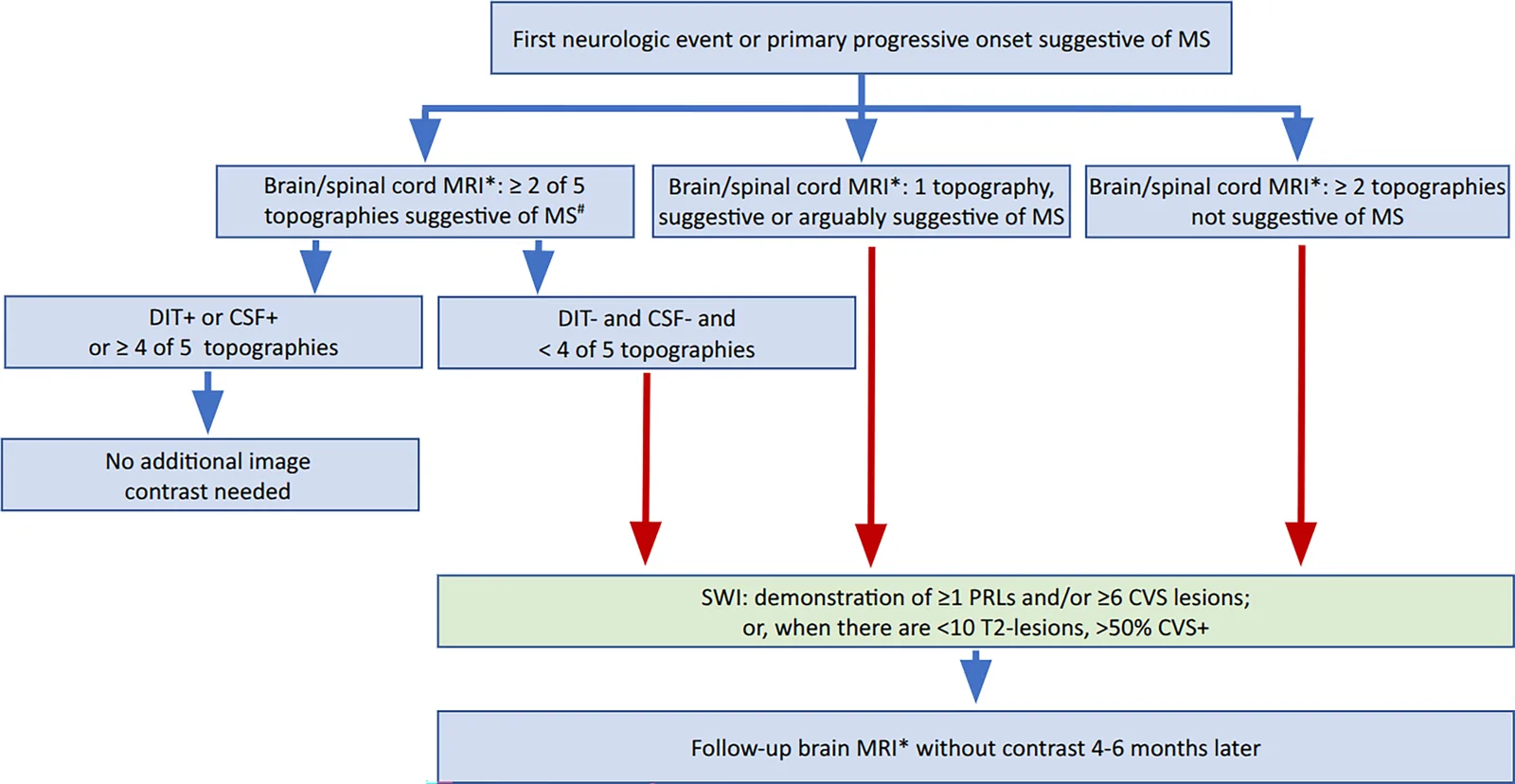
Flowchart on when SWI is needed to detect the central vein sign (*CVS*) and/or paramagnetic rim lesions (PRLs) in patients with relapsing or progressive presentation to diagnose MS according to the 2024 revision of the McDonald criteria and 2024 MAGNIMS-CMSC-NAIMS consensus recommendations. The rationale for including SWI in the initial MRI in patients with T2 lesions not suggestive of MS is that detection of a single T2 lesion in one of the five topographies suggestive of MS upon follow-up would then allow to diagnose MS. *CSF* cerebrospinal fluid, *DIT* dissemination in time. * Compliant with 2024 MAGNIMS-NAIMS-CMSC consensus recommendations [3]. # According to the 2024 revision of the McDonald criteria [2]

**Fig. 6:**
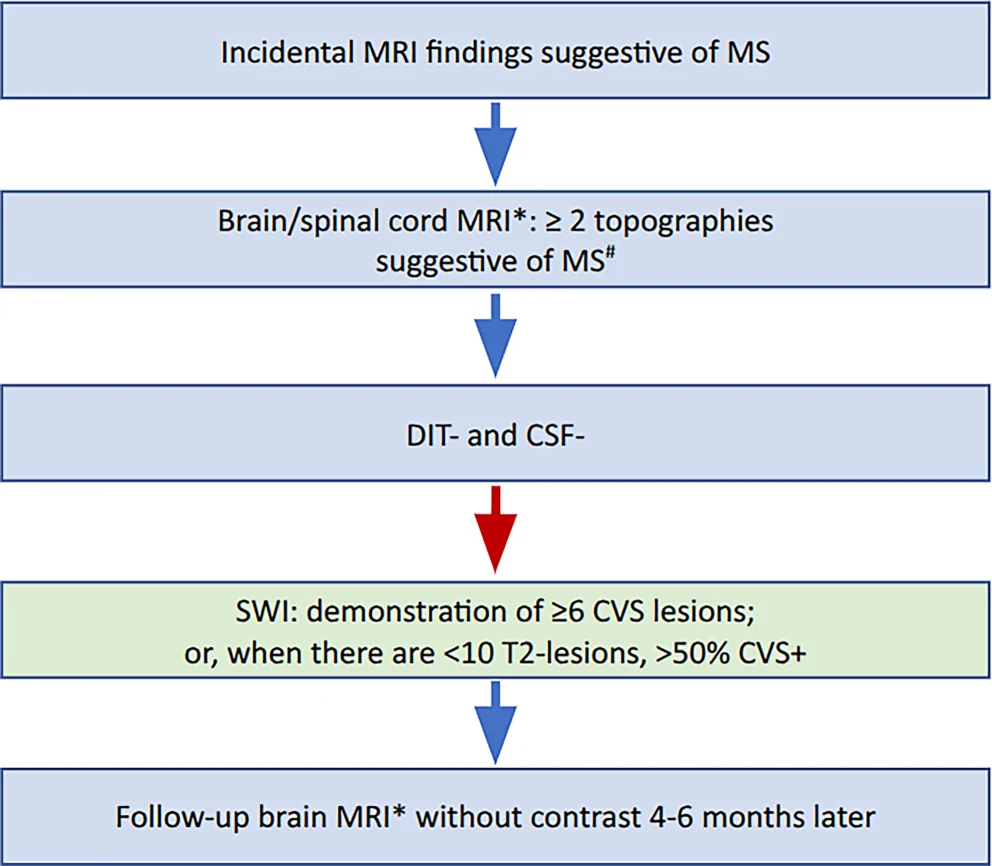
Flowchart on when SWI is needed to detect the central vein sign (*CVS*) in asymptomatic individuals or patients with non-specific presentations (i.e., neurological symptoms that do not constitute a clear typical attack or progression of disability, such as unspecific paroxysmal symptoms) to diagnose MS according to the 2024 revision of the McDonald criteria and 2024 MAGNIMS-CMSC-NAIMS consensus recommendations. *CSF* cerebrospinal fluid, *DIT* dissemination in time. * Compliant with 2024 MAGNIMS-NAIMS-CMSC consensus recommendations [3]. # According to the 2024 revision of the McDonald criteria [2]

The 2024 revision of the McDonald criteria shifts the focus toward a unified, “biological” definition of MS to facilitate earlier diagnosis. The integration of novel imaging biomarkers poses new challenges for neurologists and (neuro)radiologists in selecting efficient, resource-conscious diagnostic algorithms. Although definitive validation studies in the clinical routine setting are currently limited, it can be anticipated that established diagnostic MR acquisition protocols according to previous recommendations [12], specifically the core sequences defined in Table 1, combined with clinical history and CSF analysis, are sufficient to diagnose MS in the vast majority of patients without requiring advanced imaging biomarkers such as imaging of the ON [6, 37], CVS or PRL; Table 7 summarizes current DIS and DIT criteria. Consequently, to balance diagnostic efficacy with efficient resource utilization, we recommend a tiered diagnostic approach as outlined in Fig. 4 (ON-MRI), Fig. 5 (symptomatic patients), and Fig. 6 (asymptomatic individuals formally classified as RIS patients). In general, we propose the following workflow:

- Optic Nerve Imaging: We do not recommend ON-MRI in every patient, particularly not in those in whom the diagnosis of MS can be conclusively established using the core MRI protocol. We recommend obtaining an ON-MRI in patients presenting with typical or atypical optic neuritis (Fig. 4) when OCT and VEP are unavailable, negative, and, particularly, in cases that remain inconclusive. ON involvement provides critical data regarding potential differential diagnoses, such as NMOSD or MOGAD [36].
- CVS and PRLs: We do not recommend acquiring SWI as part of the core acquisition protocol for every patient. Instead, we propose the stratified approach detailed in Figs. 5 and 6, reserving SWI for patients in whom identifying CVS and/or PRL potentially alters the diagnostic conclusion.

**Table 7:** Dissemination in Space and Time according to [2]

**Dissemination in Space (DIS)**
*5 typical lesion topographies*
Optic nerve^1^
Periventricular
(Juxta)cortical
Infratentorial
Spinal cord
*DIS is positive when typical lesions are present in at least in 2 out of these 5 topographies.*
*In patients with progressive disease, two (or more) spinal cord lesions are sufficient to identify DIS.*
**Dissemination in Time (DIT)**
*Radiologically*
Any Gd-enhancing lesion, regardless of location/topography or timing
Any new T2-hyperintense lesion compared to a previous MRI, regardless of location/topography
Enlarging/expanding (“evolving”) lesions are currently not considered DIT evidence
CVS and PRL may support the inflammatory etiology of T2-hypertense lesions, but are, as of today, not explicitly used to discriminate lesions of non-inflammatory causes, e.g., when occurring in (older) patients with small vessel disease.
*Clinically*
Any new clinical attack upon follow-up

^1^ Newly included compared to the 2017 McDonald criteria [105], but already part of the 2016 MAGNIMS consensus guidelines [106]

In *symptomatic patients* presenting with a first clinical event or with a progressive onset (Fig. 5), the 2024 McDonald criteria allow for the diagnosis of MS even if only a single DIS topography is affected [2]. Specifically, patients with a single lesion in any of the five DIS topographies (periventricular, cortical/juxtacortical, optic nerve, infratentorial, or spinal cord) qualify for a diagnosis if PRL or CVS positivity is demonstrated alongside either CSF positivity or DIT. This highlights the use of new MRI biomarkers as substitutes for DIT or CSF positivity.

- PRL positivity: Defined as the detection of ≥ 1 PRL.
- CVS positivity: Defined by the detection of ≥ 6 CVS-positive lesions (“select‑6 rule”) or, in patients with fewer than 10 white matter lesions, when more than 50% of lesions exhibit a CVS (“majority rule”).

However, it is important to note that the original data on optimal CVS thresholds and their subsequent sensitivity and specificity vary across studies [77–79]. In symptomatic patients with ≥ 2 DIS topographies, CVS positivity is sufficient for an MS diagnosis, even in the absence of DIT and CSF findings. Similarly, in cases of clinical progression lasting at least 12 months and with two proven spinal cord lesions, CVS positivity is diagnostically sufficient for MS even in the absence of DIT and positive CSF testing.

In *asymptomatic individuals* (formally classified as RIS) or those with a presentation with symptoms not specific to MS (e.g, paroxysmal symptoms or other non-specific neurological symptoms; Fig. 6) who present with ≥ 2 DIS topographies involved, CVS positivity alone is sufficient to diagnose MS. Thus, we do recommend the use of SWI in order to demonstrate CVS+ lesions in DIS-positive RIS patients without DIT and negative CSF. Notably, current criteria include only CVS positivity (as defined above), but not PRLs, to support and establish an MS diagnosis in RIS patients, despite the fact that a single PRL suffices to relax the DIS requirement to a single topography in symptomatic patients. Furthermore, neither CVS nor PRLs have been chosen to support DIT, although their presence may be particularly useful for establishing the inflammatory nature of new (or possibly even expanding) lesions, especially in individuals aged ≥ 50 years.

In patients *demonstrating “red flags”* (such as age ≥ 50, significant vascular risk factors (e.g., hypertension, smoking, diabetes, hyperlipidemia, known macrovascular disease), or headache disorders), additional features are strongly recommended to increase specificity. These features include a spinal cord lesion, CSF positivity, or CVS positivity. If neither spinal cord lesions nor CSF positivity is detected, SWI diagnostic inclusion is strongly recommended.

Finally, the 2024 revisions of the McDonald criteria apply only to the diagnosis of MS. Unlike their 2021 precursor [12], the 2024 MAGNIMS-CMSC-NAIMS consensus recommendations do not address disease monitoring [3]. For patients requiring follow-up imaging to establish a diagnosis according to these criteria, we recommend a brain MRI without gadolinium-based contrast agents (GBCA) at an interval of 4–6 months. Follow-up spinal cord MRI is generally not recommended, as the occurrence of new asymptomatic spinal cord lesions in this context is rather infrequent [80].

### MR Imaging to Monitor Treatment Efficacy and Predict Treatment Response

MRI plays a crucial role in disease monitoring, particularly in predicting and tracking treatment efficacy and detecting comorbidities, especially in the context of pharmacovigilance.

#### Standardized Brain and Spinal Cord MRI Protocols

**Fig. 7:**
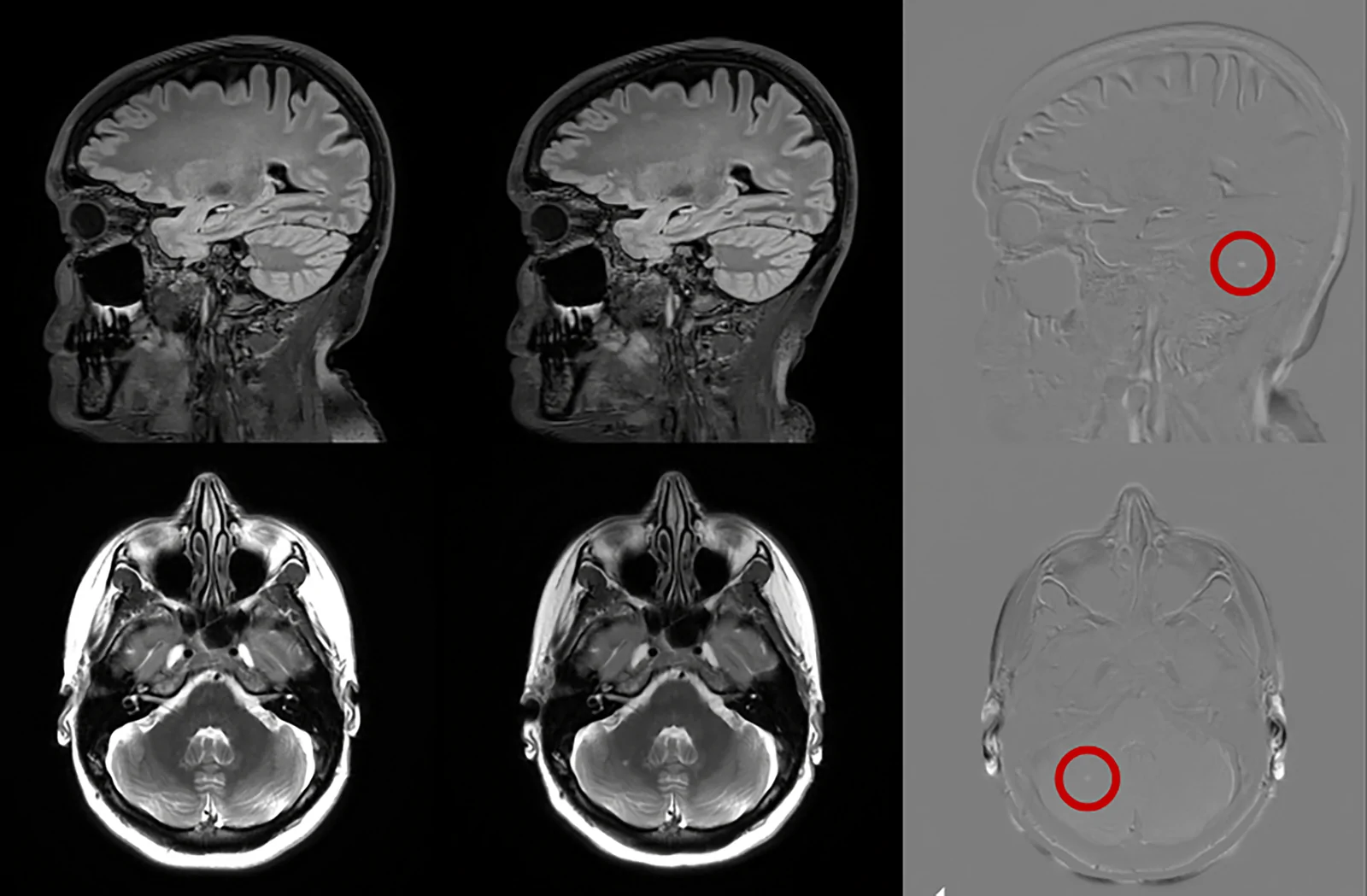
MS disease monitoring by MRI reveals a clinically silent, new infratentorial T2-hyperintense lesion on 3D T2w FLAIR and axial T2w TSE at follow-up (middle panel) compared to baseline (left panel). Consistent acquisition (same scanner and coil, same sequence parameters) of 3D sequences facilitates reliable detection of both new and enlarging T2-hyperintense lesions using longitudinal subtraction (right panel) [86]

Standardized MR image acquisition is crucial for the reliable monitoring of treatment in MS patients and should be consistent with the protocols used to establish an MS diagnosis (Fig. 7). However, in contrast to the MR acquisition used for diagnostic purposes, MR acquisition for treatment monitoring can utilise an abbreviated scan core protocol that combines 3D T2w FLAIR, including multiplanar reconstructions, and, in cases where there is a clinical need to use gadolinium-based contrast agents (GBCAs), a contrast-enhanced T1w sequence. In challenging cases or when 2D T2w FLAIR sequences or 3D T2w FLAIR sequences of insufficient quality are acquired, axial T2w (T)SE/(F)SE is recommended as part of the core monitoring protocol. Notably, the use of contrast media should be considered optional rather than mandatory for disease monitoring. The standardized brain and spinal cord MR acquisition protocols for treatment monitoring purposes are presented in detail in Table 1. The use of SWI sequences is recommended when sufficient expertise in image acquisition and interpretation is available, but it is not part of the core protocol. Along the same lines, acquiring heavily T1w sequences, such as MPRAGE, for longitudinal morphometry/volumetry is recommended only for centers with expertise. Additional pulse sequences, such as DWI, T2w DIR, and T1w PSIR, are optional and not recommended as part of the core protocol. We further do not recommend other advanced MRI techniques, including diffusion tensor imaging (DTI), functional MRI (fMRI), magnetisation transfer ratio (MTR) imaging, myelin water fraction (MWF) imaging, and imaging of certain anatomic regions, such as the optic nerve, in the clinical routine follow-up setting [32].

#### MRI Measures for the Assessment of Inflammatory Disease Activity

##### Contrast-Enhancing and Active T2 Lesions in the Brain

Contrast-enhancing lesions (CELs) in MS, detected by application of GBCAs, reflect inflammatory disease activity leading to blood-brain barrier disruption [81]. CELs have been used for decades in MS patients to demonstrate inflammatory disease activity during disease-modifying treatment (DMT), particularly to detect subclinical disease activity, as CELs are 5–10 times more sensitive than clinical relapses [23, 82]. However, the time frame over which CELs are detectable is limited to a median of 2 weeks [83, 84]. The corresponding T2-hyperintense lesion of a CEL remains visible after the resolution of contrast enhancement [84], and each CEL is considered to be accompanied by a T2-hyperintensity from the start. Therefore, new or enlarging T2-hyperintense lesions (interim active T2 lesions) are more suitable as a sensitive imaging marker of intermittent or continuing inflammatory disease activity if a technically comparable and relatively recent MRI (≤ 1 year) is available. The sensitivity for detecting subclinical disease activity based on the presence of active T2 lesions can be further increased by using longitudinal subtraction techniques (Fig. 7; [85, 86]). The time interval of brain MRI without the use of GBCA for routine treatment monitoring in clinically stable (no clinical relapse or progression) patients should not exceed 12 months. In general, according to recent guidelines, the use of GBCAs for treatment monitoring should be limited as much as possible. Following regulatory authorities’ directives and in accordance with the use of GBCAs for diagnostic purposes, a single dose (0.1 mmol/kg body weight) of a macrocyclic GBCA should be used exclusively. The use of GBCAs should be considered in cases of clinical suspicion of inflammatory disease activity, as this information can aid clinical decision-making regarding the indication for steroid treatment and the initiation or escalation of DMT. In addition, certain DMTs require the detection of inflammatory disease activity, which necessitates the identification of CELs, particularly in patients with progressive MS [87].

#### Spinal Cord Lesions

The prevalence and relevance of asymptomatic spinal cord lesions in MS patients have been underrated for a long time. In particular, in later stages of relapsing and in progressive MS patients, an acceleration of spinal cord lesion load occurs with considerable consequences for disability progression [88]. Asymptomatic spinal cord lesions may occur independently of inflammatory disease activity in the brain in approximately 10% of clinically stable relapsing MS patients [89]. Data on enlarging spinal cord lesions are mostly lacking. However, given the technical and neuroradiological challenges associated with spinal cord MRI acquisition and interpretation, this poses a risk of both, false-positive and false-negative results, potentially leading to incorrect treatment decisions (e.g., treatment escalation). As such, spinal cord MRI during follow-up is recommended only in certain clinical situations, for example, verifying spinal cord relapses or evaluating unexpected spinal cord symptoms. Therefore, spinal cord MRI for routine treatment monitoring in the clinical setting should be performed primarily by experienced centers using a robust, high-quality MR acquisition protocol. However, given the varying degrees of spinal cord involvement in MS, it remains unclear at what intervals after diagnosis or after the start of treatment the spinal cord should be examined for clinically silent disease progression or atrophy.

#### Slowly Expanding Lesions (SELs) and Paramagnetic Rim Lesions (PRLs)

SELs and PRLs are increasingly attracting attention in the context of treatment monitoring, in particular in patients with a progressive MS disease course, because these lesion types reflect tissue damage linked to chronic inflammatory activity related to certain clinical outcome measures such as disability progression [51, 90, 91]. The interpretation of SELs is based on T2w FLAIR or heavily T1w images, which show a slight progression of the spatial lesion extent over weeks, months, and years. SEL detection relies on advanced postprocessing tools, which remains a major barrier to clinical implementation so far. PRLs are reflecting lesions with a rim of activated microglia and macrophages containing myelin debris, which may slowly expand over a period of 3–5 years. PRLs are detected using iron-sensitive pulse sequences, primarily processed PHA reconstruction of SWI. Both lesion types most likely reflect histopathologically chronic active-inactive lesions, with an inactive center showing no capacity for remyelination and an active rim demonstrating ongoing chronic active (“smouldering”) inflammatory demyelination, and both types exhibit overlap in some lesions [52]. Such SEL-PRLs, i.e., the combination of the two, may be particularly precarious for disease progression and prognosis. Yet, SELs can present with or without an iron rim, and PRLs can occur without expansion [92]. In addition, in certain lesions, the morphology of the iron rim may change, or the rim may completely disappear. There is an increasing interest in using these imaging measures as markers of treatment efficacy. However, the first studies including Cladribine and Tolebrutinib failed to convincingly demonstrate any treatment effect related to the drug on the number of PRLs [93]. Given the lack of solid evidence that any approved DMTs have a significant effect on PRLs/SELs, they are so far not yet recommended as markers to monitor treatment efficacy in the clinical routine setting. SWI for treatment monitoring should be used exclusively in centers with sufficient expertise in this field and the ability to standardize image acquisition over time.

#### Cortical Grey Matter Lesions

The clinical relevance of cortical grey matter lesions for the diagnosis and prognosis of MS has been conclusively demonstrated. Similar to spinal cord pathology, cortical grey matter pathology, including focal lesions, accumulates and accelerates in later disease states, particularly in progressive MS patients [94].

However, reliable detection of cortical lesions requires the acquisition of dedicated pulse sequences, such as double-inversion recovery and/or phase-sensitive inversion recovery. Even with these techniques, sensitivity remains relatively low, and inter-rater variability in cortical grey matter lesions is high [95, 96]. In addition, the risk of inaccurate lesion interpretation, including false-positive and false-negative results, is substantial. Therefore, we do not currently recommend using MRI of cortical lesions as an imaging marker for treatment monitoring purposes.

#### Brain and Spinal Cord Atrophy

Over the past 20 years, substantial research has focused on understanding the clinical significance of brain and, more recently, spinal cord atrophy in MS and on developing reliable methods to estimate it. These advancements have expanded the options available to clinicians and researchers alike. However, despite these improvements, the reliable quantification of brain and, in some aspects, even more challenging, spinal cord atrophy in clinical practice remains elusive. Several factors contribute to this limitation, including uncertainty of individual results, a lack of comparative studies and reliable post-processing workflows, and numerous confounding factors that can lead to overestimation or underestimation, such as hydration state, lifestyle, and comorbidities [97, 98].

Another critical issue is the lack of appropriate normative databases of brain (sub-)volumes and cortical thickness for cross-sectional comparisons with MS patients. Together with the substantial variability of brain (sub-)volumes and cortical thickness across individuals, this gap complicates the quantification and interpretation of atrophy assessments and limits the applicability of these methods for clinical decision-making [97, 99]. Longitudinally, it is the speed of intraindividual atrophy evolution within patients and across that matters in MS. To assess this properly, scans recorded along the timeline must be registered to an unbiased midspace, preferably inversely consistent, to subject each time point to the same amount of interpolation and degrading. While such methods exist, these have not yet been widely implemented. In summary, while atrophy measures may help to guide treatment decisions, potential errors must be addressed, and more research is required to confirm their accuracy and reliability. Therefore, we do not recommend their use in everyday clinical decision-making, but we do recommend their application by experienced centers using highly standardized image acquisition, post-processing, and analysis protocols.

#### AI-Based Assessment of Disease

Artificial Intelligence (AI) tools designed to support the radiological workflow in MS imaging are increasingly entering the market [100]. These tools promise to enhance efficiency and quality by reducing reading time and improving reporting accuracy. Development is particularly rapid in two key aspects:

First, significant progress has been made in the automated segmentation of white-matter lesions, well exemplified in the availability of open-source algorithms that segment lesions with expert-level accuracy, including McDonald criteria-based localization, in the brain [101] and spine [102]. This has led vendors to offer commercial solutions. These aim to automatically annotate lesions, provide objective lesion load metrics (number, volume, or both), and detect new or enlarging lesions in follow-up imaging.

Second, AI has solidified brain atrophy as a viable clinical biomarker. Commercial pipelines now promise rapid, fully automated volumetry of critical structures (e.g., thalamus, cortical grey matter) to quantify neurodegeneration with much greater precision than visual assessment. However, reliable longitudinal volumetric assessment remains challenging, as discussed above; recent comparisons suggest that biological changes are often smaller in magnitude than the inherent technical measurement noise in real data, and that algorithmic variance is significant across AI models [103].

Crucially, a recent meta-review found limited evidence for the clinical value of these tools [104]. Therefore, while they may be explored on an individual basis for specific solutions, commercial AI tools cannot yet be recommended for general use. Large-scale comparative and real-world studies are still necessary to validate their utility.

#### Billing/Reimbursement of MS-MRIs in Germany

In the Federal Republic of Germany, billing and reimbursement of medical benefits are regulated by medical fee schedules for physicians. For private health insurances, this is the GOÄ (“Gebührenordnung für Ärzte”). For statutory health insurances, the uniform assessment standard (EBM = “Einheitlicher Bewertungsmaßstab”; https://ebm.kbv.de/) issues a list of numbered positions (GOPs = “Gebührenordnungspositionen”) which, along with other factors, determines final reimbursement. Here, reimbursement is distributed by the responsible regional “Kassenärztliche Vereinigung” (KV), a statutory health insurance association operating under the auspices of the “Kassenärztliche Bundesvereinigung” (KBV), to licensed medical practitioners and specialists. Reimbursement under EBM is limited by a total volume of financial compensation, whereas there is no such budget that limits GOÄ billing. At the individual physician level, EBM reimbursement is determined by standard (“Regelleistungsvolumen”, RLV) and qualification-dependent (“Qualifikationsgebundenes Zusatzvolumen”, QZV) service provisions. If MS-MRIs are provided within an Outpatient Specialist Network for MS (“Ambulante Spezialfachärztliche Versorgung für MS”, ASV-MS), which can be launched under specific conditions when various regulations are met, these will be reimbursed by extrabudgetary recompense (indicated by adding the letter “A” to the corresponding GOP, cf. Table 8).

**Table 8:** 2026 Billing codes according to GOP and GOÄ for MS-MRIs in Germany

	GOP/EBM	GOÄ
*Brain*	34410 34452 (CE)^3^	5700 (or 5735) 5731 (CE)^1^
*Optic nerve*^*1*^	34420 34452 (CE)^3^	5700^2^ 5731 (CE)^1^
*Spinal cord*^*2*^	34111 34452 (CE)^3^	5705 (or 5735)^2^ 5731 (CE)^1^
*Additional potentially applicable codes*	24211/24212 (consultation allowances) Pseudo-GOPs for GBCA (e.g., 96609)	1 (consultation allowance), 75 (report), 344 or 346 (GBCA injection manually or via injector)

If “A” is appended to the respective GOP billing code, e.g., GOP 34410A, the MRI is reimbursed on an extrabudgetary basis within an Outpatient Specialist Network for MS (“Ambulante Spezialfachärztliche Versorgung für MS”, ASV-MS)

^1^ For any additional contrast-enhanced (CE) MRI sequences recorded after GBCA injection

^2^ 5733 can be coded for additional computer-aided analyses, 5732 can be coded when coil was changed. Brain and spinal cord together are coded by GOÄ 5735 (along with 5732 & 5733 if applicable)

^3^ When at least two contrast-enhanced (CE) MRI sequences are recorded

EBM-compliant MRI examinations of the brain, ON and/or spinal cord can be conducted and billed within a single visit. The relevant positions are GOP 34410 for the brain and GOP 34411 for the cervical and thoracic spine. There is no dedicated GOP for optic nerve MRI per se but GOP 34420 (facial/viscerocranial MRI) can be used as an umbrella code. However, imaging in two planes is then mandatory. While the EBM/GOP-relevant QBK-RL (cf. supplementary material) specifies only three recorded MRI sequences as sufficient for MS and inflammatory CNS diseases, the currently effective EBM MRI preamble actually requires a minimum of four sequences to be acquired (https://www.kbv.de/documents/praxis/abrechnung/ebm/archiv/2024-1-ebm.pdf, section 34.4, p. 831). This is inconsistent. The application of gadolinium-based contrast agents (GBCAs) is billed based on additional contracts with the KV and is usually reimbursed per milliliter of GBCA injected. When at least two contrast-enhanced MRI sequences are recorded, GOP 34452 (“additional sequences”) can be billed. In brain MRI, contrast-enhanced SWI for CVS detection, along with a subsequent 3D T1-weighted scan, qualifies for GOP 34452. If applicable, consultant allowances can be billed by GOP 24211 (for 6 to 59-year-old patients) and 24212 (for patients 60 years old and beyond). Billing according to GOÄ, on the other hand, is more flexible, less restrictive, and more profitable. GOP and GOÄ billing codes for MS-MRIs are listed in Table 8.

For further remarks on quality assurance and control (QA/QC), see the supplementary material section.

Preferably, MS-MRIs should be reimbursed only when performed in accordance with approved specialist guidelines and recommendations endorsed by the relevant professional societies, and when appropriate quality is independently assured on a regular basis.

Notably, outpatient billing constraints will need to be adjusted to make MS-MRI reimbursable according to our recommendations in the German healthcare system: For the initial diagnosis of MS (and other inflammatory CNS disorders), our core brain protocol of 3D T2w FLAIR, axial 2D T2w, and 3D or axial 2D T1w with Gd (Table 2) could not be billed according to current EBM/GOP demands and would not be QBK-RL-complaint either. According to current EBM demands, four native sequences are required and one (or two) contrast-enhanced scans (“4 + 1 or 2” claim), while QBK-RL requires at least three native and one contrast-enhanced acquisition (“3 + 1” claim; cf. supplementary Table S1). For the time being, (neuro-)radiologists in the German outpatient care setting are forced to generally expand our proposed diagnostic core brain protocol by, for example, at least a GBCA-unenhanced, predominantly T1w scan (which is not part of the core protocol, cf. Table 3) for QBK-RL-compliance, and additionally by a SWI (also not part of the core protocol, cf. Table 3) to be fully EBM-compliant. Our proposed core brain protocol for MS disease monitoring (Table 9) is far too minimalistic to be billable under current EBM- and QBK-RL regulations. Similar considerations apply to ON- and spinal cord MRI, both for diagnosing and monitoring MS (Tables 2 and 9). Clearly, regulations that require expanding MRI exams beyond the essentials by unnecessary sequences amount to an unwarranted waste of resources and should be revised appropriately to facilitate cost- and time-effective imaging. For that purpose, reimbursement should be adjusted effectively to the operating expense.

**Table 9:** MRI for MS treatment efficacy monitoring and disease activity assessment

**MRI acquisition**
Baseline MRI and follow-up MRI should be performed preferably on the same MRI scanner using the identical acquisition protocol (same hard- and software). If not possible, at least the magnetic field strengths, the spatial resolution, and the choice of pulse sequences should be identical
Use of GBCAs is optional; clinical situations that may require their use include validating atypical disease activity, informing clinical decisions, and assessing suspected comorbidities. GBCAs application should be restricted as much as possible and used exclusively with single dose (no double or triple dose)
The brain MRI acquisition should be performed according to the standardized acquisition protocols (Table 1)
Core MRI protocol: 3D T2w FLAIR, and, in challenging cases, when 2D T2w FLAIR sequences are acquired, or when or when the 3D T2w FLAIR possesses limited infratentorial sensitivity, an additional axial T2w TSE; optional Gd-enhanced T1w
Additional sequences for experienced centers: SWI (PHA and SWI reconstructions)
Volumetric measure and other advanced MRI techniques are not recommended in the clinical routine setting
Spinal cord MRI is not recommended for routine monitoring of treatment
Clinical situation requiring spinal cord MRI: verification of new, atypical, or persistent disease activity in the spinal cord, spinal cord comorbidity
Core protocol for spinal cord: sagittal T2w and PD or T2w STIR, axial T2w
Use of GBCAs should be restricted as much as possible since spinal cord lesions rarely show contrast enhancement
Optic nerve MRI is not generally recommended
**MRI measures**
New (NT2L) and enlarging (ET2L) T2 lesions are recommended for detecting inflammatory activity, regardless of their topography
Slowly expanding lesions (SELs) may indicate “smouldering” MS activity, particularly when associated with PRLs (= SEL-PRLs)
Contrast enhancement indicates inflammatory disease activity and is relevant to detect in certain situations
Brain and spinal cord atrophy measures suspicious for neurodegenerative disease progression are currently not recommended for routine therapeutic clinical decision makings, but the lack of accelerated atrophy progression can be used to support (neuro-)radiological NEDA-IV upon follow up [107], i.e., there is no (neuro-)radiological evidence for inflammatory (no NT2L/ET2L) or neurodegenerative (no accelerated atrophy progression) disease activity. Focal atrophy evolution around, for example, spinal cord lesions must not be mistaken for disease activity
**Scan intervals**
Baseline brain MRI is recommended before treatment initiation, including DMT switch; GBCAs may be required according to drug label before switching to another DMT
Re-baselining brain MRI without GBCAs 6 months after treatment initiation is recommended.
Annual brain MRI without GBCAs recommended in clinically stable patients (no clinical relapses or progression)
Short-term follow-up brain MRI without GBCAs in 6–12 months once subclinical disease activity has been detected on MRI

*D* dimensional, *DMT* disease-modifying therapy, *GBCAs* gadolinium-based contrast agents, *GRE* gradient echo, *NEDA* no evidence of disease activity, *SWI* susceptibility-weighted imaging

## Conclusion and Future Perspectives

The 2024 revisions of the McDonald criteria and the accompanying MAGNIMS-CMSC-NAIMS recommendations have further strengthened the role of MRI in the diagnosis of MS and moved the field closer to a more biologically grounded diagnostic framework. At the same time, these developments increase the technical, interpretive, and organizational challenges of MRI in the clinical routine. Based on the currently available evidence, we consider conventional brain and spinal cord MRI, interpreted together with the clinical presentation and CSF biomarkers, to remain the diagnostic backbone in the vast majority of patients. Accordingly, we recommend that optic nerve MRI and susceptibility-based biomarkers, particularly CVS and PRLs, should presently be implemented in a structured, indication-driven manner rather than indiscriminately in all patients. This appears particularly important for preserving diagnostic specificity and reducing the risk of misdiagnosis while efficiently utilizing healthcare resources.

Several questions arising from our recommendations need to be addressed by future prospective studies. First, the true incremental diagnostic value of CVS and PRLs in the context of established measures, including lesion topography, CSF oligoclonal bands, and kappa-free light chains, requires validation in real-world clinical practice across scanner vendors, field strengths, and levels of reader expertise. This is particularly relevant in patients with low lesion burden, radiologically isolated syndrome, and diagnostically challenging constellations, in whom these biomarkers may be most useful but in whom false-positive interpretation may also have the greatest clinical consequences. At present, the routine acquisition and interpretation of SWI or related susceptibility-sensitive sequences cannot be uniformly recommended for all patients, given the dependence of these biomarkers on technical parameters, spatial resolution, field strength, reconstruction methods, lesion selection, and reader experience. Accordingly, harmonization of susceptibility-based imaging protocols, standardized definitions and thresholds for CVS and PRLs, and structured training for image interpretation should be regarded as major priorities. Future multicenter studies will have to determine in which clinical scenarios these techniques meaningfully improve diagnostic accuracy, how they can be integrated with existing diagnostic criteria, and what minimum technical and interpretative standards are required for their reliable use in daily practice.

Second, future studies should clarify whether advanced MRI markers of chronic active lesions can inform therapeutic decision-making in the clinical routine setting. PRLs and SELs are biologically plausible markers of smouldering inflammation and chronic active lesion pathology and have been associated with tissue destruction and disability progression. However, it is currently unresolved whether their presence, number, persistence, or evolution should influence treatment initiation, escalation, or de-escalation, or whether they can serve as reliable markers of response to current or emerging therapies. Comparable uncertainties remain for cortical grey matter lesions, brain and spinal cord atrophy, and other quantitative MRI measures, which are promising but not yet sufficiently standardized or validated for routine treatment monitoring and individualized decision-making.

Finally, the future role of MRI in MS will depend not only on biological relevance but also on feasibility, reproducibility, and cost-effectiveness in routine care. Therefore, validation studies focusing on inter-rater reliability, automated lesion detection, and AI-supported image analysis are needed. A high degree of standardization is essential for the next phase of implementing these techniques in the clinical routine, facilitating a personalized strategy for prognostic stratification and treatment decision-making. Until these data are available, we consider a standardized tiered MRI strategy to be the most appropriate approach to balance earlier diagnosis with diagnostic safety and responsible resource use.

## Supplementary Information

ESM1: Supplementary material 1

## Data Availability

No datasets were generated or analysed during the current study.
