# Supplementary material for: Expert Panel Consensus Guidelines of the German Society of Neuroradiology on the Use of Magnetic Resonance Imaging in the Diagnosis and Monitoring of Multiple Sclerosis
Source: Clin Neuroradiol. 2026 May 26;36(3):831–54. doi: 10.1007/s00062-026-01670-4 (PMC13522060; doi:10.1007/s00062-026-01670-4)
Supplement: Supplementary file 1 [file 62_2026_1670_MOESM1_ESM.docx]

**Supplement to:**

**Expert panel consensus guidelines of the German Society of Neuroradiology on the use of magnetic resonance imaging in the diagnosis and monitoring of multiple sclerosis**

*Quality Assurance and Control (QA/QC) of MS-MRIs*

Quality assurance and control (QA/QC) of MRI scans are fundamental to the accurate diagnosis and monitoring of MS and to ensure high standards of neuroradiological performance. QA / QC should entail standardized assessments and evaluation of:

-	physical properties of MRIs,

-	clinical indication for scanning and

-	radiological reporting accuracy.

Core physical MRI properties include signal-to-noise ratio (SNR), contrast-to-noise ratio (CNR), image homogeneity, blurring/smoothness, motion (within and between slices and volumes), and artifacts (especially those from various acceleration methods).

MRI requisitions and radiology reports should contain basic information to render the exam useful and should preferably be structured. The patient's clinical presentation, questions to be addressed by the ordered MRI, previous imaging records and data, and medication history are indispensable components of the scan request.

In case of initial work-ups for suspected MS, the qualified radiology report should, in one way or the other, specify number, topography and size of T2 lesions (T2Ls), and, when examined by SWI (cf. Fig. 5 in the main manuscript), presence and number of central vein signs (CVS) and paramagnetic rim lesions (PRLs). Then, it should be stated if DIS / DIT criteria are met, if there is focal or global atrophy of the brain and/or spinal cord, and the appropriate diagnostic conclusions along with differential diagnostic considerations should be derived. For follow-up and disease monitoring, number, location and size of new T2Ls (NT2Ls) and enlarging T2Ls (ET2Ls), including SELs (= slowly enlarging/expanding/evolving T2Ls) and especially SEL-PRLs, as well as evolution of focal/global atrophy should be noted, and attention must be tuned to the development of potential complications (classic or inflammatory progressive multifocal leukoencephalopathy: c/i-PML, immune reconstitution inflammatory syndrome: IRIS, simultaneous or deteriorating PML co-occurrence with IRIS: PML-s/d-IRIS, JC virus granule cell neuronopathy etc.).

As of today, there is no formal, standardized, or unified quality assessment of MS-MRIs across the various institutions providing radiological services for both outpatient and inpatient care in Germany and most, if not all, other countries worldwide. Thus, QA/QC remains, at best, limited to in-house protocols and is often rudimentary or mostly even nonexistent.

For example, SNR and CNR of MS-MRIs are, although crucial for diagnostic sensitivity, not usually controlled or evaluated, and cutoff criteria for minimally acceptable SNR / CNR metrics have not been established for broad application to MS-MRIs. Subtle protocol changes, even from simple scanning software updates, can affect physical MRI properties and hamper, or even preclude, the diagnostic utility of, for example, appropriately co-registered longitudinal within-contrast (FLAIR, PD-, T2- or T1-weighted) scan subtractions [[1,2]](https://www.zotero.org/google-docs/?m1syW3). On the other hand, advanced QA / QC scripts for structural MRI processing and evaluation have been published and are publicly available for brain [[3,4]](https://www.zotero.org/google-docs/?sRj93C) and spinal cord [[5,6]](https://www.zotero.org/google-docs/?WI61dv). These generate a range of extremely useful QA/QC metrics and can serve as templates for systematic, automated QA/QC of MS-MRIs. Our consortium explicitly encourages translating these or similar approaches into clinical practice for systematic QA/QC and data harmonization of MS-MRIs. Such future efforts should be orchestrated, and MRI vendors could support these with high impact.

Although standard criteria for MRI quality control are released by the Federal Joint Committee (G-BA = “Gemeinsamer Bundesausschuss”)^[1]^ in Germany, these apply only to statutorily health-insured outpatients. In other words, there is no and has never been any mandatory and regular MRI quality control for inpatients or privately insured outpatients in Germany. This is both regrettable and obviously not desirable. Furthermore, the G-BA has currently suspended any MRI quality control by random samples until 2030, even for statutory health insurances (<https://www.g-ba.de/beschluesse/6928/>). Random sampling for MRI QA/QC was performed by the authorized Regional Associations of Statutory Health Insurance Physicians (“Kassenärztliche Vereinigungen,” KVs), and only the current MRI was sampled; i.e., previous exams were not requested for QA/QC. Such practice is insufficient in the case of MS follow-ups, where especially NT2Ls / ET2Ls have to be assessed with respect to previous MRI examinations, as well as in the case of SELs (and now SEL-PRLs), where it may be necessary to consider not just the most recent MRI of the patient but also others dating back longer. This is laborious and currently not reimbursed.

German standard G-BA criteria for MRI quality control are issued according to § 135b para. 2 SGB V – QBK-RL (“G-BA Richtlinie über Kriterien zur Qualitätsbeurteilung in der Kernspintomographie“ = standard procedures on criteria for quality control in magnetic resonance tomography). The most recent version took effect in 2025 and can be found here: <https://www.g-ba.de/downloads/62-492-3687/QBK-RL_2024-11-22_iK_2025-01-01.pdf>. These criteria remain effective to avoid potential claims of recourse and regress indemnities.

Although the QBK-RL has been revised recently, the corresponding MRI scan specifications for MS and inflammatory CNS diseases (section 1.1.4, pp. 8+9) fall far below the standards set by the previous 2021 and the current 2024 MAGNIMS-CMSC-NAIMS [7,8] as well as our consensus recommendations laid out here, while, at the same time, formally requiring unnecessary scans and sequences (at least one native T1w is always required according to QBK-RL, also for follow-up/disease monitoring). For example, the QBK-RL does not specify a recommended field strength, and the requested spatial resolution is generally lower (e.g., 1x1x5 mm3 for 2D, 1.5 mm3 isotropic for 3D brain exams) than the consensus recommendations of the MS specialists’ associations, and there is no statement on interslice gaps. SWI for CVS and PRL detection is not part of the QBK-RL brain MS protocol, and there are no dedicated protocols for optic nerve (ON) or spinal cord MRI, according to QBK-RL. Table S1 summarizes the differences between the current 2024 MAGNIMS-CMSC-NAIMS consensus recommendations [[7]](https://www.zotero.org/google-docs/?pKW1ik) and the effective QBK-RL standards for MS-MRIs. Such disparities should be discontinued. QBK-RL protocols need to be reconciled in line with the relevant consensus recommendations. The quality of MS-MRIs should be independently audited regularly for both out- and inpatients, regardless of their statutory or private health insurance. This is crucial to ensure accurate, cost-effective diagnostic work-ups and follow-ups.

**Supplementary Table S1. Disparities between the 2024 MAGNIMS-CMSC-NAIMS consensus recommendations and the QBK-RL standards for MS-MRIs in Germany, with main differences highlighted in red. Both pertain only to the diagnosis, not follow-up or therapeutic monitoring of MS.**

|  |  | MAGNIMS-CMSC-NAIMS 2024* | QBK-RL 2025** |
| --- | --- | --- | --- |
| Brain | Field strength | ≥ 1.5 Tesla | not specified |
|  | GBCA | recommended | mandatory |
|  | Sequences | 3D preferred  core: T2-FLAIR, axial T2, SWI, CE-T1  additional: DWI, DIR or PSIR | 2D / 3D equivalent  core: Axial T2+PD (dual echo) or FLAIR + T1 + sagittal T2 [sic!] |
|  | Spatial resolution | 3D: 1x1x1 mm^3^  2D: 1x1x3 mm^3^, no interslice gap | 3D: 1.5x1.5x1.5 mm^3^  2D: 1x1x5 mm^3^, unspecified gap |
| Optic nerve^ | Field strength | ≥ 1.5 Tesla | not specified |
|  | GBCA | recommended | optional, if applied at least 2 CE planes required |
|  | Sequences | 2D or 3D  core: coronal FS-T2 or STIR, coronal CE-FS-T1  additional: axial FS-T2 or STIR and CE-FS-T1, 3D DIR (brain & ON) | 2D / 3D equivalent  core: axial & coronal or sagittal T2 with or without [sic!] FS, T1 without and with FS (presumably for CE scans) |
|  | Spatial resolution | 1x1x2-3 mm^3^ (2D / 3D not discriminated but 3D DIR for brain & ON 1x1x1 mm^3^),  no interslice gap | 2D: 1x1x3 mm^3^,  3D: 1x1x1 mm^3^,  unspecified gap |
| Spinal cord^#^ | Field strength | ≥ 1.5 Tesla | not specified |
|  | GBCA | recommended | optional, if applied at least 2 CE planes required |
|  | Sequences | core: 2 of 5 sagittal complementary sequences (T2, PD, STIR^†^, PSIR & MP[2]RAGE), axial T2-(T)SE/GRE or multiplanar MP[2]RAGE reconstructions^‡^, sagittal CE-T1  additional: axial CE-T1 | core: Sagittal & axial T1 + T2, coronar or other angulations optional,  additional: T2*, [PS]IR and FS-PD for cervical & thoracic spine; PD, T2* & T2-FS for lumbar spine |
|  | Spatial resolution | 1x1x3 mm^3^ for sagittal,  1x1x5 mm^3^ for axial,  no interslice gap | 2D: 1x1x3 mm^3^ for cervical spine,  1x1x4 mm^3^ for thoracic & lumbar spine;  3D: 1.5x1.5x1.5 mm^3^;  unspecified gap |

CE…contrast (agent) enhanced, DIR…double inversion recovery, GBCA…gadolinium-based contrast agents, GRE…gradient echo, FS…fat saturation, MP[2]RAGE…magnetization-prepared [2] rapid [acquisition] gradient echo[s], ON…optic nerve, PD…proton (spin) density, PSIR…phase-sensitive inversion recovery (or PCIR…phase-corrected inversion recovery), STIR…short tau inversion recovery, (T)SE…(turbo) spin echo

*…according to Barkhof et al. [[7]](https://www.zotero.org/google-docs/?AUaN37)

**…according to <https://www.g-ba.de/downloads/62-492-3687/QBK-RL_2024-11-22_iK_2025-01-01.pdf>

^…There is no specifically dedicated optic nerve section in QBK-RL which lumps together “orbital space-occupying and optic nerve processes, ocular muscles” under “orbit” (1.2.3).

^#^...There is no specifically dedicated spinal cord section in QBK-RL, which lumps together “spine and trunk skeleton including neural structures” under the cervical, thoracic, and lumbar spine sections (3.1.1, 3.1.2, and 3.1.3). Here, the spinal cord and medullary conus are just listed as reference structures.

^†^…STIR should not be used after GBCA application because enhancing lesions will undergo T1 shortening and may inadvertently be nulled. This pitfall is *not* mentioned or addressed in the 2024 MAGNIMS-CMSC-NAIMS consensus recommendations.

^‡^…at least covering the cervical segment

**References**

[1. Moraal B, Wattjes MP, Geurts JJG, Knol DL, van Schijndel RA, Pouwels PJW, et al. Improved Detection of Active Multiple Sclerosis Lesions: 3D Subtraction Imaging. Radiology. 2010;255:154–63. https://doi.org/10.1148/radiol.09090814](https://www.zotero.org/google-docs/?MfwDy6)

[2. Eichinger P, Wiestler H, Zhang H, Biberacher V, Kirschke JS, Zimmer C, et al. A novel imaging technique for better detecting new lesions in multiple sclerosis. J Neurol. 2017;264:1909–18. https://doi.org/10.1007/s00415-017-8576-y](https://www.zotero.org/google-docs/?MfwDy6)

[3. Alfaro-Almagro F, Jenkinson M, Bangerter NK, Andersson JLR, Griffanti L, Douaud G, et al. Image processing and Quality Control for the first 10,000 brain imaging datasets from UK Biobank. NeuroImage. 2018;166:400–24. https://doi.org/10.1016/j.neuroimage.2017.10.034](https://www.zotero.org/google-docs/?MfwDy6)

[4. Esteban O, Birman D, Schaer M, Koyejo OO, Poldrack RA, Gorgolewski KJ. MRIQC: Advancing the automatic prediction of image quality in MRI from unseen sites. PLOS ONE. Public Library of Science; 2017;12:e0184661. https://doi.org/10.1371/journal.pone.0184661](https://www.zotero.org/google-docs/?MfwDy6)

[5. De Leener B, Lévy S, Dupont SM, Fonov VS, Stikov N, Louis Collins D, et al. SCT: Spinal Cord Toolbox, an open-source software for processing spinal cord MRI data. NeuroImage. 2017;145:24–43. https://doi.org/10.1016/j.neuroimage.2016.10.009](https://www.zotero.org/google-docs/?MfwDy6)

[6. Valošek J, Cohen-Adad J. Reproducible Spinal Cord Quantitative MRI Analysis with the Spinal Cord Toolbox. Magn Reson Med Sci MRMS Off J Jpn Soc Magn Reson Med. 2024;23:307–15. https://doi.org/10.2463/mrms.rev.2023-0159](https://www.zotero.org/google-docs/?MfwDy6)

[7. Barkhof F, Reich DS, Oh J, Rocca MA, Li DKB, Sati P, et al. 2024 MAGNIMS-CMSC-NAIMS consensus recommendations on the use of MRI for the diagnosis of multiple sclerosis. Lancet Neurol. 2025;24:866–79. https://doi.org/10.1016/S1474-4422(25)00304-7](https://www.zotero.org/google-docs/?MfwDy6)

1. The G-BA committee is formed by the Federal Association of Statutory Health Insurance Physicians (KBV = “Kassenärztliche Bundesvereinigung”), the German Hospital Federation (DKG = “Deutsche Krankenhausgesellschaft”) and the Central Federal Association of Statutory Health Insurances (“GKV Spitzenverband”).
